# Sensory modality of initiation cues modulates action goal-relevant neural representations

**DOI:** 10.1162/IMAG.a.57

**Published:** 2025-06-26

**Authors:** Nicholas Kreter, Neil M. Dundon, Jolinda Smith, Michelle Marneweck

**Affiliations:** Department of Human Physiology, University of Oregon, Eugene, OR, United States; Department of Psychological and Brain Sciences, University of California Santa Barbara, Santa Barbara, CA, United States; Department of Social and Psychological Sciences, University of Huddersfield, UK; Institute of Neuroscience, University of Oregon, Eugene, OR, United States; Phil and Penny Knight Campus for Accelerating Scientific Impact, Eugene, OR, United States

**Keywords:** reaching, neural representations, multisensory, fMRI

## Abstract

The ability to produce goal-directed movement relies on the integration of diverse sensory cues which contribute to goal subtasks, such as trajectory planning and initiation. To achieve these subtasks, the central nervous system must flexibly weight sensory contributions depending on task context, and sensory cues from one modality may contribute to multiple subtasks. Neural representations of goal-relevant features are flexible to a variety of sensory contexts. It remains less clear how neural representations of goal-relevant features are modulated when initiation-relevant features rely on sensory cues from overlapping sensory modalities. We used Bayesian pattern component modeling of fMRI data during a delayed reach task with either visual or audiovisual go-cues to explore whether neural representations of goal-related features in sensorimotor areas are modulated by changes to initiation-relevant sensory information. We found that representations of gaze direction and target direction in the primary sensory areas, motor areas, and posterior parietal cortex varied depending on whether a reach was cued with a visual or audiovisual go-cue. These findings suggest that the central nervous system flexibly delegates the tasks of ‘where’ to move and ‘when’ to move based on available sensory context, even if initiation-relevant stimuli provide no additional information about target location.

## 1 Introduction

The ability to perform goal-directed movements is essential to many behaviors in daily life. Successful planning and execution of these actions necessitates integrating rich sources of sensory stimuli in support of movement subtasks, such as tracking when to initiate a movement in the presence of an external cue (Jerjian et al., 2020), and localizing the position and orientation of the necessary effector relative to a target location (Beurze et al., 2007;Medendorp et al., 2005). For instance, the simple goal-directed action of reaching to a phone in response to an incoming call may involve contributions from visual, proprioceptive, and auditory systems. The visual system helps to identify the phone’s location in space relative to the body, the proprioceptive system helps identify the position and orientation of the hand that will be used to reach, and if the phone is ringing; the auditory system will provide information about the spatial location of the phone and an external cue that the reach should occur.

Despite their critical role for planning and execution of actions, sensory inputs are inherently noisy. The central nervous system (CNS) optimally integrates sensory inputs from multiple sources to reduce uncertainty and achieve perceptual and motor goals (Berniker & Kording, 2011;Deneve et al., 2001;Körding & Wolpert, 2006). In perceptuomotor tasks, when multiple sensory cues are available to perform the same task, the brain flexibly weights the contribution of sensory sources depending on their reliability (Berniker & Kording, 2011;Ernst & Banks, 2002;Körding & Wolpert, 2006). For instance, representations of goal-relevant features, such as a reach target’s direction, change depending on the sensory context in which they are presented (Beurze et al., 2007,^2010^;Medendorp et al., 2005). Visual, auditory, superior parietal, dorsal premotor, and primary motor areas show distinct goal-relevant representations of reach targets that are presented with visual (Beurze et al., 2007;Mcguire & Sabes, 2009;Medendorp et al., 2005;Sober & Sabes, 2005), auditory (Cohen & Andersen, 2000;Cohen et al., 2002;Mullette-Gillman et al., 2005), or proprioceptive (Bernier & Grafton, 2010) cues, suggesting that sensorimotor regions are flexible to changing sensory contexts of goal-related sensory information. This framework, and its evidence, suggest that the CNS is flexible to weight sensory information of goal-relevant task features. In turn, this flexibility is reflected in sensory-specific goal-relevant representations. While it is well established that the brain can combine input from different senses when they support the same part of an action (i.e., both vision and audition providing cues to locate a reach target), it is less clear how the CNS flexibly weights multisensory information that supports different parts of the same action (i.e., visual cues contribute to target localization and initiation while auditory cues contribute only to initiation). Under models of Bayesian inference, sensory inputs should be assigned to each subtask based on their relative reliability and availability for task demands.

Externally cued motor tasks often rely on sensory systems not only for goal-relevant information (i.e., where or how to move), but also for initiation-relevant information (i.e., when to move, absent of spatial information). Previous work has suggested that movement initiation and planning are functionally independent processes within the sensorimotor system (Haith et al., 2016;Wong et al., 2017), and movement initiation can be observed in sensorimotor regions independent of the action being prepared (Kaufman et al., 2016). While initiation and planning are functionally independent, the two subtasks rely on overlapping regions within the CNS and may receive the same sensory inputs. In real-world settings where cues for initiation and planning come from several modalities, the brain must selectively allocate sensory resources based on each modality’s availability and reliability for the corresponding action subtask. For instance, the CNS will differentially weight visual input when it supports multiple tasks (Hayhoe & Rothkopf, 2011;Sprague et al., 2007) compared to when some tasks can be offloaded or shared with an additional sensory modality (e.g., an audio and visual cue for movement initiation). In turn, the weighting of sensory inputs for initiation, will define the best available sensory resources for goal-relevant task features. Thus, goal-relevant representations may be modulated by the extent to which initiation-relevant information is provided by unimodal or multiple sensory modalities—we test this hypothesis for the first time here. During fMRI, participants performed a visually guided reach to target task across two sessions where movement initiation was cued 1) with a separate visual stimulus, and 2) with an audiovisual stimulus. Consistent with our hypothesis, results showed that goal-relevant task representations were modulated by the number of sensory initiation cues, suggesting a flexible CNS that differentially allocates sensory resources based on their reliability to achieve motor goals.

## 2 Materials and Methods

### 2.1 Participants

Twenty-five healthy young adults [16 f, mean (SD) = 21.1 (2.7) years] were recruited from the local community and provided informed consent to participate in this IRB approved study. Exclusion criteria included self-reported left-handedness (or as determined by the Edinburgh handedness inventory), neurological diagnosis, neuromuscular injury, and self-reported motor or cognitive impairment that would adversely impact the ability to perform the experimental reaching tasks.

### 2.2 Materials, design, and procedures

**Fig. 1.**
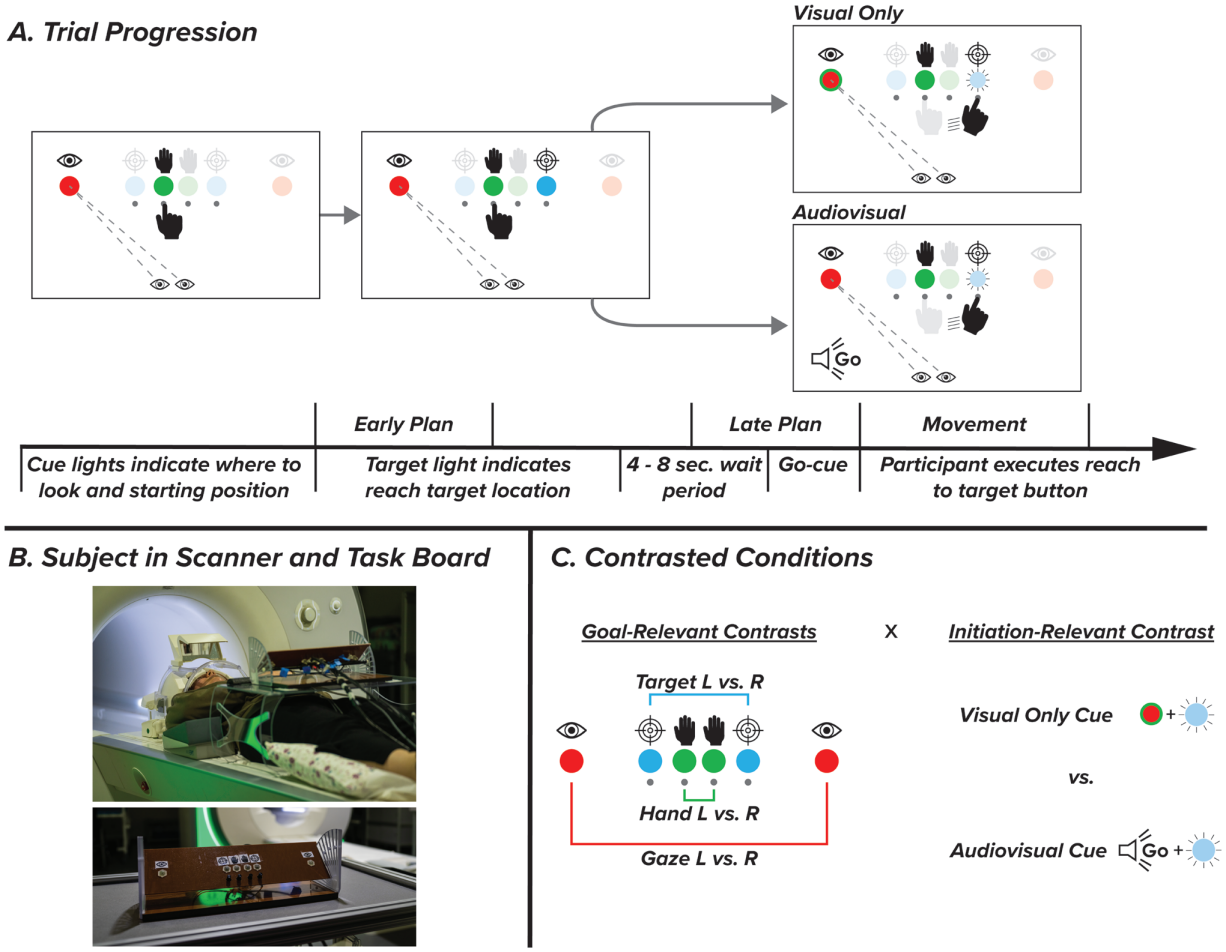
Experimental design and contrasts. (A) Participants performed eight trial types with either left or right gaze, left or right initial hand position, and a left or right target. Trials started with a gaze light instructing participants where to look and a hand light instructing which button to start pressing with their right hand. Once pressing the correct button, a target light would appear. After a variable wait period of 4–8 s, participants received either a visual or audiovisual go cue and reached to the button under the target light. General linear models were computed to predict BOLD activity during the first 2 s of the wait period (early plan) the final 2 s of the wait period (late plan), and movement initiation. (B) Participants performed the task on an interactive board while laying in the scanner. A mirror attached to the head coil allowed participants to see each of the buttons, lights, and their hand. (C) Contrasts tested the interaction between goal-relevant (gaze, target, and hand direction) and initiation-relevant (initiation-cue modality) task features, as well as individual main effects of direction and modality.

Subjects completed a goal-directed reaching task using a custom-built task board during two scanning sessions. Session order was counterbalanced across subjects to ensure no effect of condition order. Sessions were performed on separate days and each session featured six functional runs. Structural imaging was performed during each participant’s first session. The task board featured LED lights that indicated the starting hand position, gaze direction, and reach target (Fig. 1). Eight unique conditions were used that featured a combination of left or right initial hand position, gaze direction, and reach target direction. Participants performed these conditions across two sessions where only the sensory modality of the go-cue differed: one session featured trials with only visual go cues while the other featured an audiovisual go cue. During trials with the visual-only go cue, the gaze light changed color, and the target light turned off simultaneously. The trials with audiovisual go cues featured an auditory beep at the same time as the target light turned off.

Each trial began when the LED indicating hand position and gaze direction illuminated. After participants pressed the correct hand-position button there was a variable delay period, followed by a variable plan period where a third LED indicated the reach target. The variable delay period was randomly drawn from a selection of [1, 2, 4, 8, or 16 s] with respective proportions of trials [0.52, 0.26, 0.13, 0.06, 0.03] while the plan period was randomly drawn from [4, 6 or 8 s] with respective proportions of [0.56, 0.30, 0.14]. The variable intertrial delay period served to minimize the preceding trial’s movement-related activity bleeding into the next trial’s planning period. Following the plan period, participants received a cue to reach towards the target. At the onset of the movement initiation cue, the hand and target light disappeared synchronously with the change to the color of the gaze LED (visual session), or the auditory cue playing to both ears through headphones (audiovisual session). Following completion of the reach to target button, an audio cue played through headphones indicating success or failure. If the wrong target button was pressed, or no movement was made for 2.5 s after the go cue, the trial was counted as a failure. Each session featured six functional runs of 40 trials, with breaks between runs. In this study, a subset of trials featured a no-go stimulus where participants were instructed not to perform a reach, but data from these no-go trials are not presented in this manuscript. All events from stimulus presentation to button lift and button press timing were controlled with a custom Python script.

The order of trials and durations of the delay and plan periods were determined to minimize the variance inflation factor (VIF) within each functional run (Ariani et al., 2022). VIF estimates the degree of multicollinearity in a model by providing an index of how much the variance of a single regression coefficient increases due to collinearity. VIF values close to 1.0 indicate no correlation between regressors whereas larger VIF values indicate that regressors are not independent of each other. In the present study average VIF was around 1.15, which indicates the independence of regressors (Ariani et al., 2022).

The dimensions of the task board were 40.0 cm wide, 10.0 cm long, and 6.5 cm deep. It consisted of the six LED lights indicating gaze, hand, and reach target locations, as well as four buttons beneath the hand and target LEDs. Icons indicating the gaze, hand, and target LEDs were placed above each LED. The LEDs indicating left and right-hand position were located 2.3 cm apart at the midline of the board. Each target and gaze LED was 2.3 and 6.9 cm lateral to each hand LED, respectively. Throughout testing, the task board was positioned on a stand 4 cm above the surface of the table and angled at 40.6° relative to the table surface. Pilot testing indicated that this position and angle were optimal for participants to maximize their view of the task. After receiving task instructions, but before being placed in the scanner, each participant performed practice trials prior to familiarize themselves with the experimental task.

All trials in this experiment were performed during fMRI in a Siemens Skyra (Siemens Medical, Germany; 32-channel phased-array head coil). During the first session, imaging began with a high-resolution T1 weighted anatomical scan (TR/TE = 2500/2.98 ms, flip Angle = 7°, FOV = 256 mm). Following the anatomical scan, participants performed the reaching task while BOLD contrast was measured with a multiband T2*-weighted echoplanar gradient echo imaging sequence (TR/TE = 450/30 ms, flip Angle = 45°, FOV = 192 mm; multiband factor: 6). Each full brain functional image consisted of 48 slices acquired parallel to the AC-PC plane (3 mm thickness; 3 x 3 mm in-plane resolution) (Moeller et al., 2010;Setsompop et al., 2012). To minimize the effects of motion, padding was added to secure the head, neck, and shoulder for all participants. The goal-directed reaching task was performed on an interactive board placed at arm’s length on a marked spot on a table spanning each participants’ hips. With a mirror attached to the head coil, participants could see the task board and their hand as if they were viewing them while sitting upright.

Pilot eye tracking data (*n*= 2) was collected outside of the scanner to ensure that participants could maintain a constant fixation on the gaze light while still performing the reach task. Pilot participants completed a block of 40-trials with their head secured in a headrest. Eye trackers (Pupil Core Eye Tracking Platform, Pupil Labs) monitored gaze behavior with an eye camera resolution of 192 x 192 pixels and a sampling frequency of 200 Hz. Raw gaze data were filtered to eliminate blinks and gaze values with confidence values below 0.6. Pupil Player Software (v3.5.1) was used to determine gaze accuracy by calculating the proportion of time where fixations stayed within a 3.5 x 3.5 cm box surrounding the instructed left or right fixation lights. Pilot participants maintained accurate fixations on the correct gaze lights 99.3% of the time. Direct gaze tracking was not performed in the scanner; however, we predicted eye movement using the recently developed deep neural network model, DeepMREye (Frey et al., 2021). We calculated changes in gaze position using DeepMREye x- and y- gaze position coordinates. Participants maintained the correct gaze direction for 97.9% (SD = 2.2) of trials during session one (visual-only go cue) and 98.0% (SD = 1.4) of trials during session two (audiovisual go cue). Paired t-tests revealed no differences between sessions (p = 0.848).

### 2.3 Data processing and statistical analysis

MRI pre-processing and analysis were performed with SPM12 (Welcome Trust Center for Neuroimaging, London, UK), FSL (Jenkinson et al., 2012), and custom MATLAB (Natick, MA) code. Using 2^nd^degree B-spline interpolation in SPM, subjects’ functional images were spatially realigned to a mean image. Images were then coregistered to the T1-weighted image and normalized. Mean head motion rotations and translations were minimal (minimum and maximum values in parentheses): x: 0.015 mm (-2.681, 1.714); y: 0.108 mm (-1.886, 3.754); z: 0.215 mm (-3.922, 4.188); pitch: -0.0002° (-0.1471, 0.1509); roll: 0.0008° (-0.0365, 0.0387); yaw: 0.0013° (-0.0941, 0.0495). Functional images were each inspected for distortions and inhomogeneities and trials were excluded if they featured greater than 2 mm translation within a single run. Trials were removed for only one subject. We also used the rWLS toolbox (Diedrichsen & Shadmehr, 2005) in SPM to account for motion artifact and down weight images with higher motion artifact.

Following pre-processing of the anatomical and functional imaging data, we assessed spatial patterned activity in pre-determined sensorimotor regions of interest (ROIs), including primary visual (V1) and auditory (Heschl) areas, superior parietal areas 5 (SPL5) and 7 (SPL7), PMd, and primary motor area 4a from the Julich brain atlas (Amunts et al., 2020). All ROIs were selected from the left hemisphere to capture activity related to the control of the contralateral (i.e., right) hand. Spatial patterned activity differences were assessed between conditions in the ROIs by implementing Bayesian representational similarity analyses (vRSA) (Friston et al., 2019). Convolution-based general linear models were run for each of the eight experimental conditions from each session. To account for the temporal progression of representations throughout planning and execution (Cappadocia et al., 2017;Churchland et al., 2006;Hadjidimitrakis et al., 2014,^2022^;Messier & Kalaska, 2000), two general linear models were used to separately assess event-based activity during planning and movement, respectively. The general linear model for planning focused on event-based activity in an “early-planning” epoch, starting at target light presentation and lasting for 2 s, as well as a “late-planning” epoch, start 2 s before the go-cue and ending at movement onset. For each epoch within this model there was one regressor for each combination of initial gaze direction (left or right), target direction (left or right), and hand position (left or right) (i.e., 16 regressors total, eight for each epoch). Movement-related activity was modeled separately as a regressor of no interest. The general linear model for the movement epoch focused on event-based activity starting at movement onset and lasting until movement completion (i.e., 8 regressors. One for each of the eight conditions). In the movement model, planning-related activity was modeled separately as a single regressor of no interest. For both models, errors (i.e., inaccurate reach trials), and no-go trials were modeled separately as regressors of no interest. For the movement model, movements between the end of a trial and the start of the following trial (i.e., moving the hand to the next starting hand position) were modeled as a regressor of no interest. The two general linear models were run for each scanning session, resulting in eight betas per epoch, per session. Betas from the two sessions were later concatenated into a single activation map (seeSupplementary Document) so that task features related to planning (i.e., target direction, gaze direction, hand position) and initiation (i.e., cue modality) could be analyzed within the same vRSA model. Planning and movement events were split into two general linear models instead of one to avoid violating the assumption of non-multicollinearity. Custom Matlab code to run vRSA analysis is publicly availablehttps://github.com/dundonnm/vRSAb.

Variational representational similarity analysis (vRSA) compared between-condition dissimilarity in spatial activity patterns in sensorimotor ROIs through a method that decomposes second-order statistics. The analysis procedure starts with a 16-row condition-by-voxel matrix (U) for each participant. These 16 rows cover two initial hand positions (left vs. right), two gaze directions (left vs. right), two target directions (left vs. right) and two cue modalities (visual vs. audiovisual). This U-matrix accounts for the mean voxel activity pattern during each condition, as calculated by the general linear model. Note, as recommended by the original published procedure, each row of the U-matrix is demeaned to be zero centered so that condition-specific magnitude differences are removed (Friston et al., 2019). Nevertheless, U-matrix beta distribution maps for key interaction contrasts—see below—can be found in theSupplementary Document. Next, we construct a 16-by-16 second-order similarity matrix (G = UU^T^) where the main diagonal represents the covariance explained by each condition. In this G-matrix, greater off-diagonal values indicate greater pattern similarity between conditions. Following the construction of the G-matrix, we can then test specific hypotheses by examining the contribution of components (i.e., effects) to G. Components included the initial hand position (left vs. right), gaze direction (left vs. right), target direction (left vs. right), initiation cue modality (visual vs. audiovisual), and the two-way interactions between hand, gaze, target location, and cue modality, respectively. Three-way interactions were included in the analyses but are not presented as they produced no evidence of a meaningful effect. Four-way interactions were not included. Visual representation of the ROIs and specific contrasts can be found inFigure 2. vRSA analyses were used to test the same hypotheses in early-plan, late-plan, and movement epochs.

**Fig. 2.**
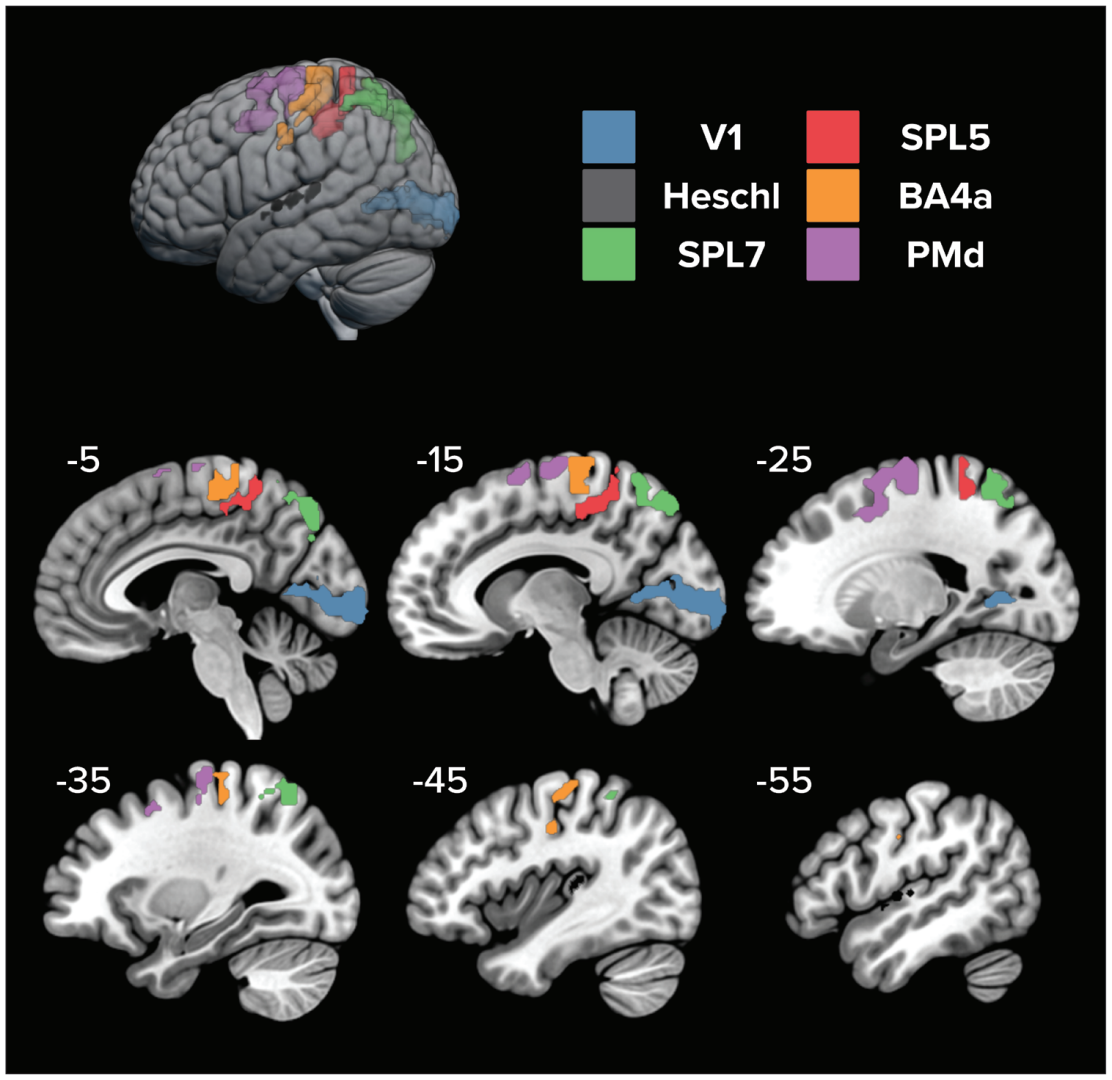
Predefined regions of interest from the Julich Brain Atlas are displayed on the MNI-152 atlas using MRIcroGL software. V1: primary visual, Heschl: Heschl’s gyrus, SPL: superior parietal lobule, PMd: dorsal premotor, BA4a: primary motor.

A strength of vRSA is assessing the independent contribution of each component to the covariance matrix G while controlling for each of the other contrasted conditions. For example, if we observe an effect of cue modality in a specific ROI, that finding is true irrespective of whether the participant was planning to reach to a left or right target. Further, interaction effects in this analysis show whether changes in spatial patterned activity from one effect are dependent on changes to a second effect, irrespective of changes in each of the other effects. For example, a credible cue modality by gaze-direction interaction indicates that spatial patterned activity differences between left and right-sided gaze conditions vary depending on the sensory modality of the initiation cue. In other words, gaze direction representations are modulated by audiovisual and visual initiation cues (regardless of whether the hand or target was on the left or right). Any interaction between goal-relevant and initiation-relevant effects would confirm our hypothesis (i.e., sensory initiation cue × target direction, sensory initiation cue × gaze direction, or sensory initiation cue × initial hand position). These interactions would support the hypothesis that changes to initiation-relevant sensory cues modulate the goal-relevant representations of reaching. While the individual effects testing target direction, gaze direction, hand position, and cue modality do not directly test our hypothesis, we include them in our model because previous literature suggests existent representations in the tested sensorimotor ROIs. A specific benefit of the vRSA method is the ability to test individual hypotheses related to the contribution of a single effect, or interaction effects, while controlling for all other component effects included in the model comparison framework.

Bayesian vRSA analyses performed using SPM return log evidence values of a hypothesis-specific representation at the group level. Log evidence values that are more negative indicate greater evidence of a credible effect. To ensure sufficient log evidence is observed, we performed an additional permutation analysis with 1000 iterations where condition labels were shuffled randomly. Calculating a G-matrix for each iteration and testing the shuffled data for the same main effects and interactions results in a null distribution for each contrast. Each observation in this permutation test is essentially a new experiment where we can assume that the null hypothesis is true. With this distribution of null observations, we can empirically build and test our actual data against a null distribution. That is, we subtract the actual (unshuffled) log evidence from the log evidence of each of the observations in the null (shuffled) distribution to create a distribution of log Bayes factors for each contrast, where higher values now communicate strong effects. We report whether the 95% highest density interval (HDI; analogous to a confidence interval) of the distribution of log Bayes factors is greater than log(3). Effects that meet these criteria are taken as being strongly credible results. In other words, we assume substantial evidence if the real data is three times more likely than the top 5% of data from the null distribution.

Our statistical methods adopt several distinct features to account for issues related to multiple comparisons. First, we use a hierarchical model with the same set of default priors to test each of the contrasted effects in parallel. Therefore, each model is an independent test of the pattern composition of an ROI. Second, for each ROI we performed a permutation analysis to create a null distribution of evidence specific to each contrast. While typical Bayesian analyses compare H1 relative to H0, this permutation analysis allows us to compare the H1 from the actual data, to the H1 of 1000 permutations of null data. We only conclude there is substantial evidence for condition-specific activity within a region if the H1 from the actual data is greater than the top 5% of data from the null-distribution. This step is a conservative addition, not typically included in vRSA analyses and diminishes the need for additional corrections due to multiple comparisons. SeeMarneweck et al. (2023)for a comprehensive discussion on how this conservative approach mitigates the need for multiple comparison corrections within and across ROIs (also seeGelman & Carlin, 2017;Gelman et al., 2012;Kruschke & Vanpaemel, 2015;Marneweck & Grafton, 2020).

Three behavioral measures were used to assess performance in the reaching task. Reach accuracy is the percentage of total reaches for which the participant hit the correct target button. Reaction time is the time between the go-cue and the participant’s finger lifting from the initial hand position button. Reach duration is the time between the participant lifting their finger from the initial hand position button and the participant pressing the correct target button. Paired t-tests were used for each behavioral measure to test differences between means in audiovisual and visual go-cue conditions. All behavioral tests were assessed for significance at α = 0.05.

## 3 Results

We used Bayesian pattern component modeling of fMRI data to assess whether goal-relevant representations in sensorimotor ROIs varied depending on initiation-relevant sensory cues during a goal-directed reaching task. Results largely supported our hypotheses that the sensory modality of initiation-relevant cues modulates goal-relevant representations in sensorimotor ROIs, with subtle changes during early-planning, late-planning, and movement epochs. Specifically, we found broad evidence for goal-relevant representations of reach target location and gaze direction to vary depending on whether movement initiation cues were visual or audiovisual. However, for the effect of hand position, we found no evidence of goal-relevant representations in sensory or motor areas and no interaction between the effects of hand position and cue modality throughout planning and movement epochs. These changes to spatial patterned activity with different initiation cues were independent of behavioral differences between trials with visual initiation cues versus those with audiovisual initiation cues.

### 3.1 Gaze, target, and hand interactions with initiation cue modality

**Fig. 3.**
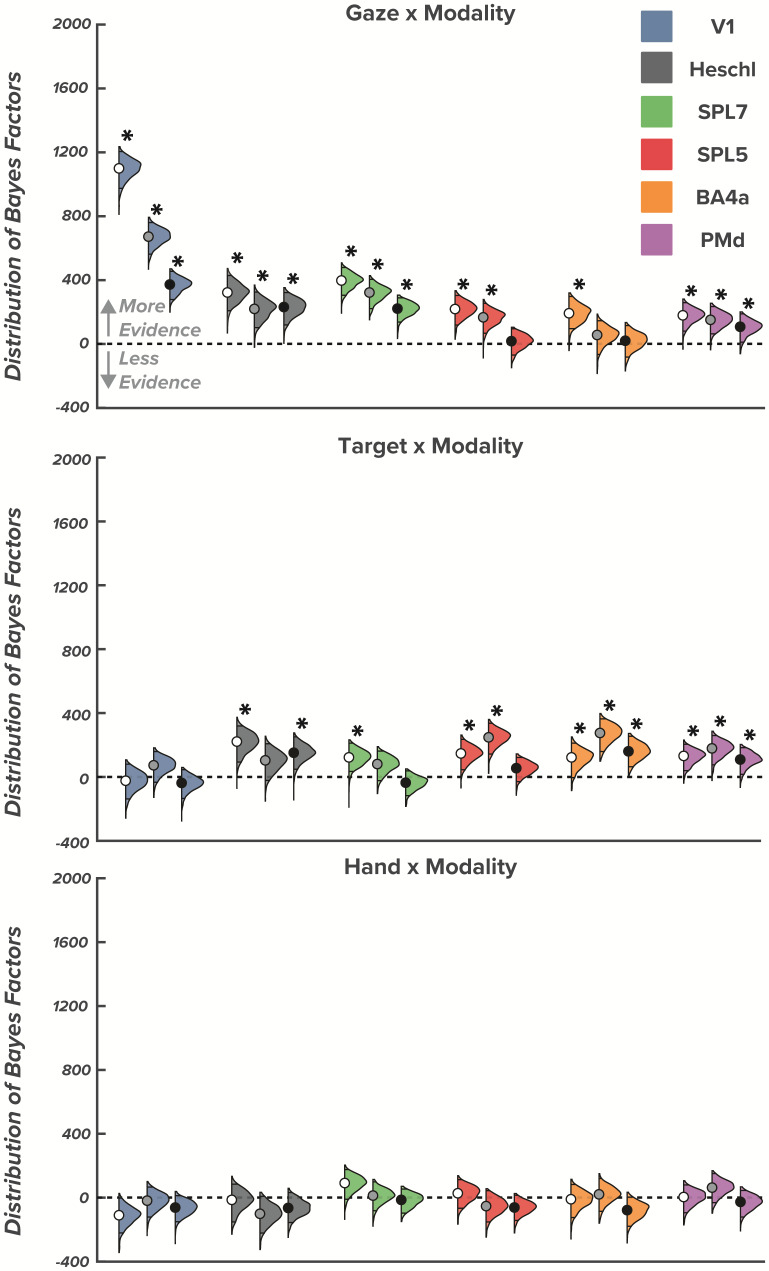
Interaction effects between goal-relevant (gaze, target, and hand direction) and initiation-relevant (cue modality) effects across early plan, late plan, and movement epochs. Violin plots show the distribution and 95% highest density intervals (HDI; dark-shaded region) of Bayes factors for the interactions between goal-relevant and initiation-relevant task features for each region of interest. Asterisks indicate substantial evidence that an effect is 3 times more credible than the 95% strongest effect from the null distribution in a specific region of interest. For each triplet of colored distributions, the left most (white dot) represents the early plan epoch, the middle (grey dot) represents the late plan epoch, and the right most (black dot) represents the movement epoch.

All regions showed effects for initial gaze direction and initiation cue modality, indicating that spatial pattern activity is distinct for left versus right gaze directions when movement initiation is cued visually versus audiovisually, respectively. We found stable, strongly credible gaze × cue modality interactions in all ROIs [P(log BF>log(3)) ≥ 0.99] during the early planning epoch (Table 1), late planning (except BA4a) (Table 2), and during movement execution (except BA4a and SPL5) (Fig. 3;Table 3). The interaction between gaze direction and initiation cue modality indicates that differences in the patterned spatial activity between left and right gaze directions vary based on the modality of the go-cue.

**Table 1.** Probability of credible evidence for each main effect and interaction during the early-plan phase for each ROI.

	Hand	Gaze	Target	Modality	Hand x Modality	Gaze x Modality	Target x Modality
ROI	**P(BF>log(3))**						
V1	0.66	**1.00**	**1.00**	**1.00**	0.01	**1.00**	0.35
SPL5	**0.98**	**1.00**	**1.00**	**1.00**	0.67	**1.00**	**1.00**
SPL7	0.93	**1.00**	**1.00**	**1.00**	0.94	**1.00**	**0.98**
BA4a	**1.00**	**1.00**	**1.00**	**1.00**	0.42	**1.00**	**0.96**
PMd	**0.98**	**1.00**	**1.00**	**1.00**	0.52	**1.00**	**0.99**
Heschl	0.74	**1.00**	0.58	**1.00**	0.40	**1.00**	**1.00**

ROIs in which P(BF>log(3)) exceeds 0.95 indicates that there is strongly credible evidence for distinct representations and are noted in bold font.

**Table 2.** Probability of credible evidence for each main effect and interaction during the late-plan phase for each ROI.

	Hand	Gaze	Target	Modality	Hand x Modality	Gaze x Modality	Target x Modality
ROI	**P(BF>log(3))**						
V1	0.79	**1.00**	**1.00**	**1.00**	0.35	**1.00**	0.90
SPL5	0.57	**1.00**	**1.00**	**1.00**	0.14	**0.99**	**1.00**
SPL7	**0.98**	**1.00**	**1.00**	**1.00**	0.60	**1.00**	0.91
BA4a	0.77	**1.00**	**1.00**	**1.00**	0.65	0.81	**1.00**
PMd	0.84	**1.00**	**1.00**	**1.00**	0.88	**1.00**	**1.00**
Heschl	0.94	**1.00**	**0.96**	**1.00**	0.03	**1.00**	0.93

ROIs in which P(BF>log(3)) exceeds 0.95 indicates that there is strongly credible evidence for distinct representations and are noted in bold font.

**Table 3.** Probability of credible evidence for each main effect and interaction during the movement phase for each ROI.

	Hand	Gaze	Target	Modality	Hand x Modality	Gaze x Modality	Target x Modality
ROI	**P(BF>log(3))**						
V1	0.11	**1.00**	**0.97**	**1.00**	0.07	**1.00**	0.15
SPL5	0.11	**1.00**	**1.00**	**1.00**	0.04	0.62	0.84
SPL7	**0.98**	**1.00**	0.91	**1.00**	0.34	**1.00**	0.14
BA4a	0.29	**0.95**	**1.00**	**1.00**	0.02	0.64	**1.00**
PMd	0.59	**0.99**	**1.00**	**1.00**	0.25	**0.97**	**0.98**
Heschl	0.49	**1.00**	0.90	**1.00**	0.09	**1.00**	**0.99**

ROIs in which P(BF>log(3)) exceeds 0.95 indicates that there is strongly credible evidence for distinct representations and are noted in bold font.

Similar to the gaze × cue modality interaction, a target × cue modality interaction indicates that the differences in spatial patterned activity for changes in target location (left vs. right) are modulated with different go-cue modalities. While gaze × cue modality interactions were relatively stable throughout plan and move epochs, we found sensory initiation cues modulate target position representations broadly during early planning, with strongly credible target × cue modality interaction in all ROIs [P(log BF>log(3)) ≥ 0.96] except for V1 [P(log BF>log(3)) = 0.35], with somewhat increasing selectively from late planning onwards predominantly in motor-centric areas (late planning: SPL5, PMd, BA4a; movement: Heschl, PMd, BA4a) (Fig. 3). While evidence was weaker for these interactions in SPL7 and Heschl during late planning, it was still 3x stronger than the 90% strongest effect from the null distribution. Overall, the observed interactions between initiation cue modality and gaze direction, and target direction, suggest that representations of goal-relevant factors in reaching tasks are dependent on initiation-relevant factors, even if those factors provide no additional information about the required movement.

When examining hand × cue modality interactions, we found no evidence of meaningful interactions in any region for any of the three epochs, indicating that representations of initial hand position were not modulated by initiation cue modality [P(log BF>log(3)) ≤ 0.94 for all regions]. A lack of differential modulation by visual or audiovisual initiation cues suggests that initial hand position representations that are less goal-relevant than target and gaze representations can be supported by the proprioceptive system, relying less on visual and/or auditory systems.

### 3.2 Distinct representations for initiation cue modality

**Fig. 4.**
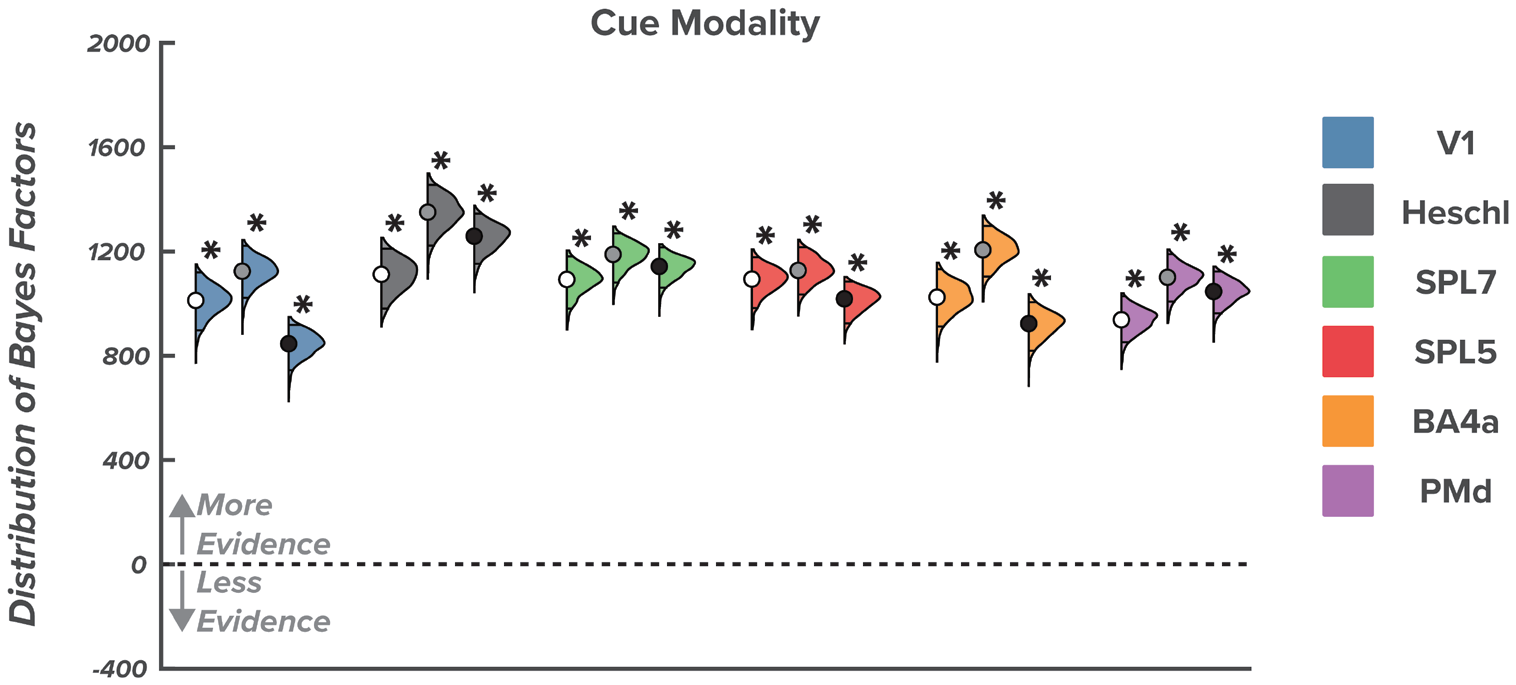
Effect of initiation cue modality across the early plan, late plan, and movement epochs. Violin plots show the distribution and 95% highest density intervals (HDI; dark shaded region) of Bayes factors for the initiation-relevant effect of cue modality for each region of interest. Asterisks indicate substantial evidence that an effect is 3 times more credible than the 95% strongest effect from the null distribution in a specific region of interest. For each colored triplet of colored distributions, the left most violin plot (white dot) represents the early plan epoch, the middle violin plot (grey dot) represents the late plan epoch, and the right most violin plot (black dot) represents the movement epoch.

All ROIs showed distinct spatial pattern activity between trials with visual versus audiovisual go-cues for each of the three planning and movement epochs [P(log BF>log(3)) = 1.00 for all ROIs]. The modulation of representations to changes in initiation cue modality that we observed in all ROIs indicates that the reaches with visual go cues were represented differently than reaches with audiovisual go cues. These results support our hypothesis that sensorimotor regions encode initiation-relevant task features (Fig. 4).

### 3.3 Representations for hand position, gaze direction, and target location

**Fig. 5.**
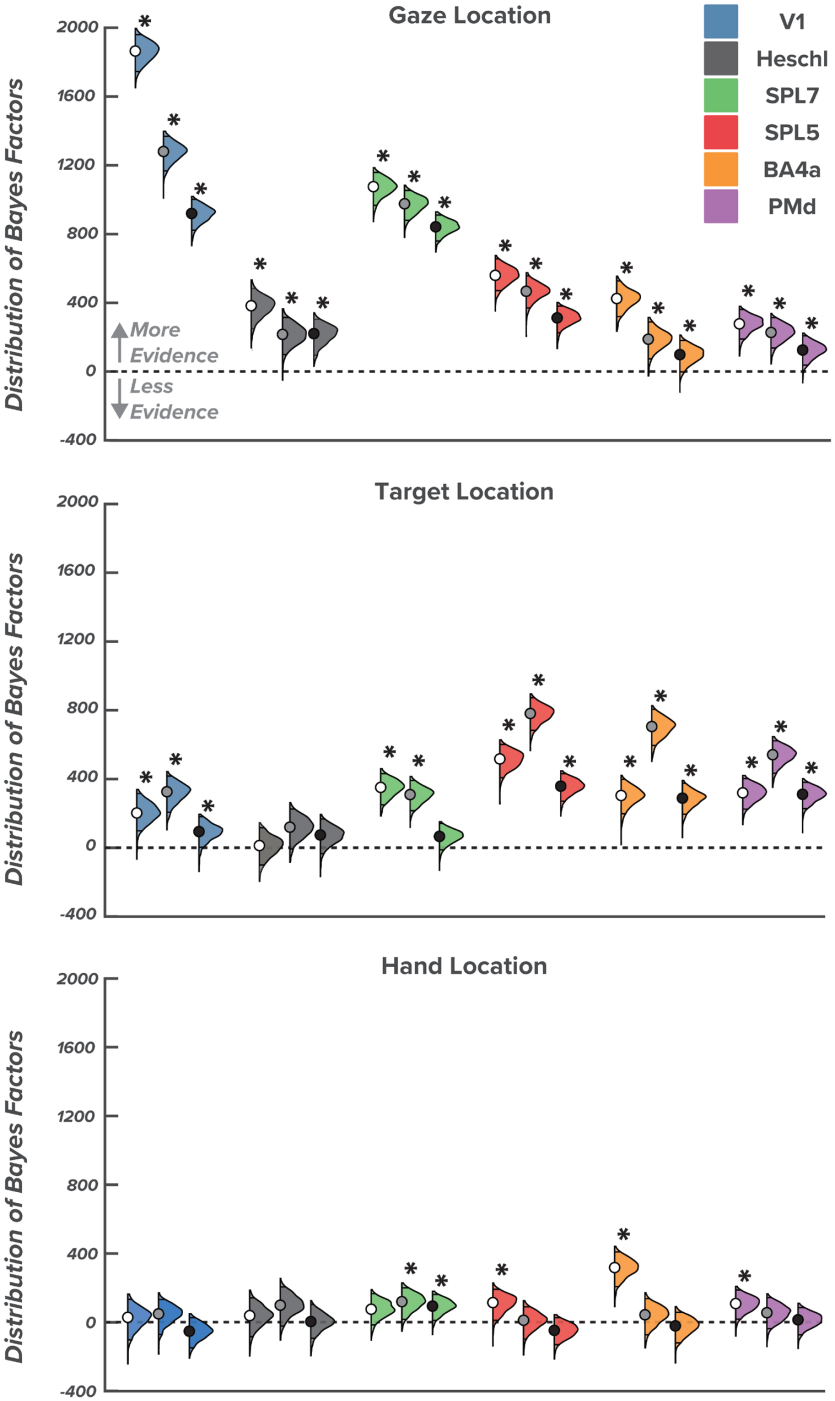
Effect of gaze, target, and hand during the early plan, late plan, and movement epochs. Violin plots show the distribution and 95% highest density intervals (HDI; dark shaded region) of Bayes factors for the goal-relevant effects of gaze, target, and hand direction, for each region of interest. Asterisks indicate substantial evidence that an effect is 3 times more credible than the 95% strongest effect from the null distribution in a specific region of interest. For each triplet of colored distributions, the left most (white dot) represents the early plan epoch, the middle (grey dot) represents the late plan epoch, and the right most (black dot) represents the movement epoch.

The effect of initial hand position was dependent on analysis epoch. During early planning, regions SPL5 [P(log BF>log(3)) = 0.98], BA4a [P(log BF>log(3)) = 1.00], and PMd [P(log BF>log(3)) = 0.98] showed distinct spatial pattern activity between trials with left and right starting hand position (Fig. 5). SPL7 was the only ROI to show a distinct representation of initial hand position during late planning [P(log BF>log(3)) = 0.98] and movement epochs [P(log BF>log(3)) = 0.98].

All ROIs were showed strongly credible effects for changes in gaze direction [P(log BF>log(3)) > 0.95 for all regions] for each of the epochs. With the exception of Heschl’s gyrus [P(log BF>log(3)) = 0.58], all other ROIs gave credible evidence for distinct representations of reach target direction during early planning. During the late planning epoch, all ROIs encoded reach target direction [P(log BF>log(3)) > 0.99]. During the movement epoch, all regions encoded reach target direction with the exception of Heschl’s gyrus [P(log BF>log(3)) = 0.90] and SPL7 [P(log BF>log(3)) = 0.91]. The vast sensitivity to changes in gaze and reach target direction both before and during movement indicates distinct representations in these ROIs for left and right gaze, and left and right targets, respectively. Critically, while these contrasts do not directly test our hypotheses, including these components allows evaluating contributions of components that test our hypotheses independent of gaze, hand position, and target direction effects that are known to drive activity pattern differences in these regions (Beurze et al., 2007;Medendorp et al., 2005;Rosenbluth & Allman, 2002;Trotter & Celebrini, 1999).

### 3.4 Differences in accuracy, duration, and reaction time with unimodal and multimodal go-cues

Participants were accurate during 97.9% (SD = 2.3%) of trials in the visual only condition and 97.9% (SD = 1.5%) of trials in the audiovisual conditions. Mean reach duration was 0.33 s (SD = 0.12) for visual only trials and 0.36 s (SD = 0.14) for the audiovisual condition. Subject showed very similar reaction times between the two testing sessions. In the audiovisual session we observed a mean reaction time of 0.762 s (SD = 0.195). In the visual-only session we observed a mean reaction time of 0.765 s (SD = 0.153). Paired t-tests revealed no difference between conditions for accuracy (p = 1.00), reach duration (p = 0.420), or reaction time (p = 0.947). Unlike previous literature, we did not observe a change in reaction time between conditions with visual and audiovisual go cues (Hecht et al., 2008;Todd, 1912). The divergence with existing literature may stem from not explicitly instructing participants to move as quickly as possible once they received a go cue. Further, many reaction time studies use simple button press tasks, whereas we used a reach task with visual cues occurring in the visual periphery. Our results suggest that changes to spatial patterned activity for target and gaze direction with audiovisual and visual cues, respectively, are not driven by behavioral differences between trials with visual initiation cues versus those with audiovisual initiation cues.

## 4 Discussion

This study investigated if goal-relevant representations in sensorimotor reach regions are modulated by the extent to which initiation-relevant information is provided by unimodal or multimodal sensory cues. During fMRI, participants performed a visually guided reach to target task across two sessions where movement initiation was cued 1) with a separate visual stimulus, and 2) with an audiovisual stimulus. Consistent with our hypothesis, results showed that goal-relevant task representations were modulated by the number of modalities contributing to sensory initiation cues, suggesting a flexible CNS that differentially allocates sensory resources based on their reliability to achieve motor goals.

A rich body of literature supports models with evidence of goal-relevant representations that are modulated by sensory context (Alaerts et al., 2009;Andersen et al., 1997;Bernier & Grafton, 2010;Girondini et al., 2024;Giurgola et al., 2024;Norris et al., 2024). We extend these findings by investigating for the first time the effect on goal-relevant representations from manipulating the number of sensory modalities cueing the initiation of movement. In real-world externally cued motor tasks, humans often depend on multiple sensory inputs not only for goal-relevant information but also for initiation-relevant information. Under these conditions, sensory resources are to be shared for goal- and initiation-relevant subtasks. The CNS differentially weights visual input when supporting multiple tasks than when it can share or offload some tasks to a different sensory modality (Hayhoe & Rothkopf, 2011;Sprague et al., 2007). In turn, the weighting of sensory inputs for initiation, will define the best available sensory resources for goal-relevant task features. Thus, representations of goal-relevant task features may be modulated by the extent to which initiation-relevant information is provided by unimodal or multimodal sensory cues (Jackson et al., 2025), which is what our results show.

In the visual-only unimodal condition, the visual system has multiple demands: gaze fixation, relative to the target, and movement initiation. Goal-relevant and initiation-relevant information must be processed and relayed from overlapping visual areas to visually responsive parietal and premotor neurons. The audio-visual multimodal condition places less demand on the visual system (movement initiation can be monitored by or in collaboration with the auditory system). That is, goal-relevant information can be relayed by the visual dorsal stream, whereas initiation-relevant information can travel via the dorsal auditory stream (Goodale & Milner, 1992;Rauschecker, 2018). Critically, the goal-relevant task demand of fixating gaze provided spatial coding information to localize the target. This task feature is present in both audiovisual and visual conditions. Thus, the experimental manipulation is specific to the number of modalities that cue initiation, varying the extent to which this subtask can be under shared control, which drives the interactions that we observed. These interactions suggest that goal-relevant representations are modulated by the extent to which sensory resources can be shared between modalities for movement initiation.

These findings potentially contribute to existing theories of Bayesian inference by showing that the addition of a redundant sensory cue can reshape the neural representations supporting action goals. Recent work has indicated that the subtasks of planning and initiation are functionally independent processes within the sensorimotor system (Haith et al., 2016;Wong et al., 2017), and neural signatures of initiation and planning can be observed in the same sensorimotor ROIs independent of one another (Kaufman et al., 2016). Despite being functionally independent subtasks, both initiation and planning are represented in overlapping regions and rely on overlapping sensory inputs to be successful. Our results show the availability of multiple sensory modalities across movement subtasks (e.g., planning vs. initiation) can lead to changes in the action goal representations in sensorimotor regions, even when behavioral performance remains constant. In contrast to traditional studies of multisensory integration (Berniker & Kording, 2011;Körding & Wolpert, 2006), which show a Bayesian process that improves perceptual estimates of action goals (e.g., target location), this finding raises the possibility that the brain may perform Bayesian inference for each subtask to optimize how sensory resources are distributed across subtasks. This speculative interpretation that the brain flexibly controls action subtasks based on available sensory context should be tested further with more causal research methods, such as TMS.

The interaction between gaze direction and cue modality was relatively stable across epochs, suggesting that the sensorimotor system encoding gaze position is broadly attuned (in space and time) to the sensory initiation cues. Notably, these effects persist throughout the movement, despite the demands on vision now matching in the unimodal and multimodal conditions. This suggest that the sensorimotor system does not update or re-allocate sensory resources unnecessarily (because the outcome has already been planned), at least not in the case of a relatively rapid, predictable simple reach to target movement.

While gaze × cue (and the lack of hand × cue) interactions were relatively stable throughout plan and move epochs, we found sensory initiation cues modulate target position representations broadly during early planning in sensory association, premotor and motor areas (Heschl, SPL7, SPL5, PMd, BA4a), and with somewhat increasing selectivity from late planning onwards predominantly in motor-centric areas (late planning: SPL5, PMd, BA4a; movement: Heschl, PMd, BA4a). That said, evidence was weaker for these interactions in SPL7 and Heschl during late planning, but still 3x stronger than the 90% strongest effect from the null distribution. We interpret the mostly persisting target × cue interaction effects in SPL5, premotor and motor areas similar to the gaze × cue interactions, in that they are driven by number of task demands under shared control between sensory modalities. Why these interaction effects between target and cue dissipate during late planning and movement in higher association areas are unclear. One hypothesis, aligned with a hierarchical model of motor control, is that by late planning, premotor and motor representations of targets, that are tuned to sensory context, are sufficient, at least under predictable task conditions. Additionally, during the movement phase, the target LED turning off when it cued movement. When the light turns off, there is no longer a physical manifestation of the reach target for the participants to reference, and results in target location being converted from an allocentric to an egocentric remembered spatial cue (Chen et al., 2011;Chen & Crawford, 2020;Elliott & Madalena, 1987;McIntyre et al., 1997). Previous work investigating reaches to remembered targets indicates that humans convert physical remembered spatial cues from an allocentric to an egocentric reference frame at the first opportunity, but these representations fade within seconds (Chen et al., 2011;Chen & Crawford, 2020;Elliott & Madalena, 1987;McIntyre et al., 1997). Given the predictability and reliability of target location in the present task, the lack of representation in sensory areas during the movement epoch may simply reflect that they are contributing less when target locations are no longer physical.

A lack of a hand position by cue modality interaction further supports the conclusion that the number of sensory initiation cues interact specifically with goal-relevant representations. Here, we used visual and audiovisual cues to prompt movement initiation, rather than a cue with proprioceptive stimuli. Individuals can monitor the position/orientation of their effector with visual and proprioceptive sensory information (Botvinick & Cohen, 1998;Graziano, 1999). In the present study, the shift from visual to audiovisual did not introduce or alleviate sensory noise from proprioception. That V1 and Heschl areas showed no evidence for representations of hand position, or hand × cue interactions during early planning, late planning, or movement epochs suggests that initial hand position representations might rely less on the visual and auditory systems. If go cues had been delivered with a combination of proprioceptive and visual information, thereby creating a conflict between tracking the go cue and tracking the effector position, we may have observed an interaction due to the increased uncertainty associated with competing proprioceptive tasks. An alternative, though not mutually exclusive, explanation for a lack of a cue modality × hand position effect is that the initial hand position is less goal-relevant than a visual target or gaze direction. As a result, the interaction with initiation cue modality is more salient for representational task features (i.e., target and gaze) that are more directly tied to the action goal. Future work should explore how different varieties of initiation-relevant stimuli interact with goal-relevant representations of movement.

Existing frameworks of Bayesian inference support sensory context-dependent activation patterns that are highly dimensional. However, it is important to note that we also found evidence of generalized representations, particularly for task features and interactions that were not directly goal-relevant. As highlighted above, representations of hand position did not interact with cue modality. Given that neither factor in this interaction is directly goal-relevant (i.e., neither starting hand position nor cue modality inform target location), but both are still useful for the task, they may be represented in a more generalized manner to simplify coding demand (Bernardi et al., 2020). Generalized representations emerge from linear mixed selective representations (Bernardi et al., 2020), which simplify the coding demand associated with task features and allows them to be shared across contexts. These representations demand less precision and are useful for tasks such as memory or learning (Ballard et al., 2019;Baram et al., 2021;Bernardi et al., 2020;Minxha et al., 2020). However, real-world tasks demand a much higher coding capacity than abstract forms allow, particularly for goal-relevant features that ultimately determine task success. Variably reweighting sensory contributions to goal-relevant task features may limit these types of generalized representations when demands for precision are higher. This process demands the recruitment of non-linear mixed selectivity representations which do not respond to stimuli in a linear manner and have far greater coding capacity (Fusi et al., 2016;Rigotti et al., 2013). Our results, which feature a mixture of distinct and generalized representations, indicate the reach system may use both forms to maintain a high degree of flexibility and precision.

Understanding the complex interactions of sensory and motor pathways in the production of goal-driven movement may provide benefits for individuals with neurologic disorders or insults. For instance, individuals with Parkinson’s Disease (PD) show altered movement initiation during both reaching (Fasano et al., 2022) and locomotor tasks (Delval, Tard, et al., 2014;Lu, Huffmaster, Tuite, Vachon, & MacKinnon, 2017), and poor ability to use visual information to plan precision stepping tasks (i.e., a goal-directed reach task with the lower limbs) (Vitório et al., 2014,^2016^). Experiments using external cues have shown improvements in movement execution with external sensory cues (Burleigh‐Jacobs, Horak, Nutt, & Obeso, 1997;Delval, Moreau, et al., 2014;Dibble et al., 2004;Lu et al., 2017;Nieuwboer, 2008;Rogers et al., 2011) and improvements in movement preparation with external auditory cues (Delval, Moreau, et al., 2014; Delval, Tard, et al., 2014), even if those cues do not provide specific spatial information (Lu et al., 2017). Our results showing that goal-relevant representations are modulated by the extent to which sensory resources can be shared between modalities for movement initiation largely align with these clinical findings, with the caveat that we did not observe changes to motor performance. The absence of a change in performance in the present study is likely because the task likely did not tax the motor system of healthy young adults, whereas in clinical gait tasks may be more demanding and carry greater risk (i.e., a fall). To our knowledge, there has been limited study of the effects of multimodal cues on feedforward gait behavior in persons with PD. While speculative, supplying a multimodal sensory cue at gait initiation may provide a level of redundancy that reduces the burden on the visual system and improves behavior. Future work should explore how representational similarity with different sensory cues changes in populations with neuromotor deficits, such as persons with PD.

One potential limitation of this study relates to the shorter distance between the two starting hand positions than the two target and two gaze positions. Parietal and motor areas are modulated by changes in gaze and target (Andersen et al., 1997;Beurze et al., 2007,^2010^;Buneo & Andersen, 2012;Cohen et al., 2002), similar to what we showed here, and effector distances during reaching tasks (Fabbri et al., 2012;Graziano, 1999;Pesaran et al., 2006). The left- and right-hand positions were separated by 2.3 cm, whereas target and gaze locations were separated by 6.9 and 16.1 cm, respectively. It is unlikely, that this small distance between left- and right-hand positions explain why we observed few distinct neural representations for the effect of starting hand position and no interaction between hand position and cue modality. First, we found widespread modulation to changes in hand position during early planning, consistent with previous work (Hadjidimitrakis et al., 2014,^2022^;Messier & Kalaska, 2000). Second, distinct representations for left and right starting hand position were found in region SPL7 during late planning and movement epochs, possibly due to its role in organizing sensory information from a variety of sensory reference frames (Andersen et al., 1997;Buneo & Andersen, 2012;Sober & Sabes, 2005). Third, other studies have seen effects with similar distance reaches (Bernier & Grafton, 2010;Beurze et al., 2010;Fabbri et al., 2012). Thus, it is likely that the direction of initial hand position is less represented than goal-relevant target and gaze positions in the lead-up to movement initiation. The narrow orientation of the hand and target buttons was selected to limit movement and allow participants to reach from the hand to the target buttons without requiring movement of the entire arm. We also chose target and gaze positions such that targets on all trials would always fall within an observer’s near peripheral zone within which there are little changes in visual acuity (Larson & Loschky, 2009). In this way, we minimized between-condition activation pattern differences related to visual acuity differences.

Another potential limitation also stemming from a design choice might have influenced a lack of a behavioral difference between audiovisual and visual conditions, unlike that shown in previous multisensory facilitation studies. In those studies, the audiovisual conditions are the sum of stimuli from distinct auditory and visual conditions (Hecht et al., 2008;Todd, 1912). Our audiovisual condition featured two initiation cues: a visual initiation cue (i.e., the target light) and an audio-initiation cue. In our design, we opted to match the number of initiation cues between audiovisual and visual conditions, such that any activation pattern differences were driven only by a difference in the initiation cue modality rather than the number of cues given to initiate the movement. Thus, like the audiovisual condition, the visual condition featured two visual initiation cues (i.e., the gaze light and the target light). Adding an auditory cue to the visual condition would have created an imbalance between the total number of initiation cues in our contrasted conditions (i.e., two for the visual condition and three for audiovisual). We suspect that such a methodological choice likely would have elicited reaction time differences due to multisensory facilitation. However, it would also have complicated the interpretation of patterned neural activity as any differences could have been attributed to the change in the quantity of initiation cues or the modality of initiation stimuli. The present design allowed us to directly test how pattern differences change in the presence of initiation cues from visual versus auditory and visual channels. With the lack of behavioral differences, we can rule out gaze light-related demands differentially interfering with spatial coding between audiovisual and visual conditions. The distinct representations we observed may also be explained by the gaze light changing color in the visual condition but not the audiovisual condition. Visual areas are modulated by both auditory stimuli and color stimuli (Conway et al., 2010;Conway & Tsao, 2006;Lueck et al., 1989). However, the pathway responsible for processing color passes from V1 through the inferior temporal cortex (Conway et al., 2010;Matsumora et al., 2008;Zeki et al., 1991). Sensorimotor and superior temporal regions, where we also observed these interactions, are more commonly associated with polysensory stimuli (Bremmer et al., 2001;Lewis & Van Essen, 2000) than simple color changes (Conway et al., 2010;Lueck et al., 1989). Finally, we did not collect gaze data throughout the experiment and could not definitively exclude trials with improper fixations. However, we demonstrated through pilot testing that participants were capable of keeping gaze fixated on the required LED light, and used deep neural network models to ensure that visual behavior was comparable between audiovisual and visual conditions.

## 5 Conclusion

Altogether, our results show that representations of the task features of goal-directed reaching differ depending on the sensory modality of initiation-relevant information. Representations of visual task features, such as gaze direction and target location, were particularly sensitive to changes between visual and audiovisual cueing. Future work should examine the effects of other multimodal go-cues, such as combined proprio-visual or proprio-auditory, on the task feature representations of goal-directed reaching, and also how different groups with neuromotor deficits incorporate initiation-relevant information for goal-directed tasks.

## Supplementary Material

Supplementary Material

## Data Availability

All data necessary to replicate the analysis will be made available in BIDS format upon reasonable request. All codes required to perform the analysis in this manuscript are available on GitHub athttps://github.com/dundonnm/vRSAb.
