# Supplementary material for: Sensory modality of initiation cues modulates action goal-relevant neural representations
Source: Imaging Neurosci (Camb). 2025 Jun 26;3:IMAG.a.57. doi: 10.1162/IMAG.a.57 (PMC12319815; doi:10.1162/IMAG.a.57)
Supplement: Supplementary Material [file imag.a.57_supp.pdf]

Supplementary Methods and Results

1) *General linear model-derived beta distribution maps for each of the selected regions of interest*

Due to our hypotheses focusing on patterned activity in reach regions, our primary analysis relied on Bayesian variational representational similarity analyses (vRSA). While log evidence yielded from such models can test hypotheses, it does not provide qualitative information about the patterns of activity entering the models. To provide a clearer picture of the covariance patterns being assessed in each of the vRSAs, we developed general linear model-derived beta distribution maps detailing the differences in voxel activation for the effects of gaze direction, hand position, target direction, and cue modality. Representative beta distribution maps are reported for each of the three epochs, and all six ROIs from the original analysis. Included are beta distribution maps that have been averaged across the same sample of participants as the primary analyses. Group-averaged data (Sections 1a-1c) show the overall trend across participants; however, participants can be different in terms of the precise activation patterns that each condition generates despite those conditions creating comparable pattern dissimilarities. Averaging across participants risks removing these effects which are captured by the vRSA. Therefore, we also present representative data from a single participant (Sections 1d-1f). We hypothesized that pattern differences between left and right gaze directions and target directions would be modulated by whether cues were either visual or audiovisual in nature. The final section contains representative G-matrices for each of the three epochs.

a) Effect of Gaze Direction and Cue Modality – Group Average

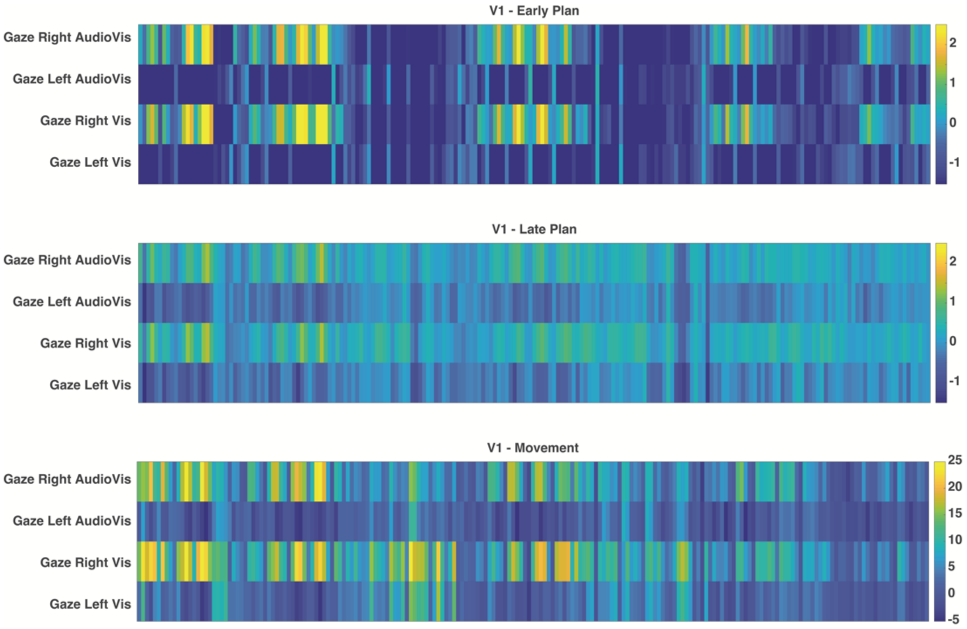

Supplementary Figure 1: General linear model-derived beta distribution maps for the effects of gaze direction and cue modality in region V1. Each row in the map is a condition and each column is a voxel in the ROI.

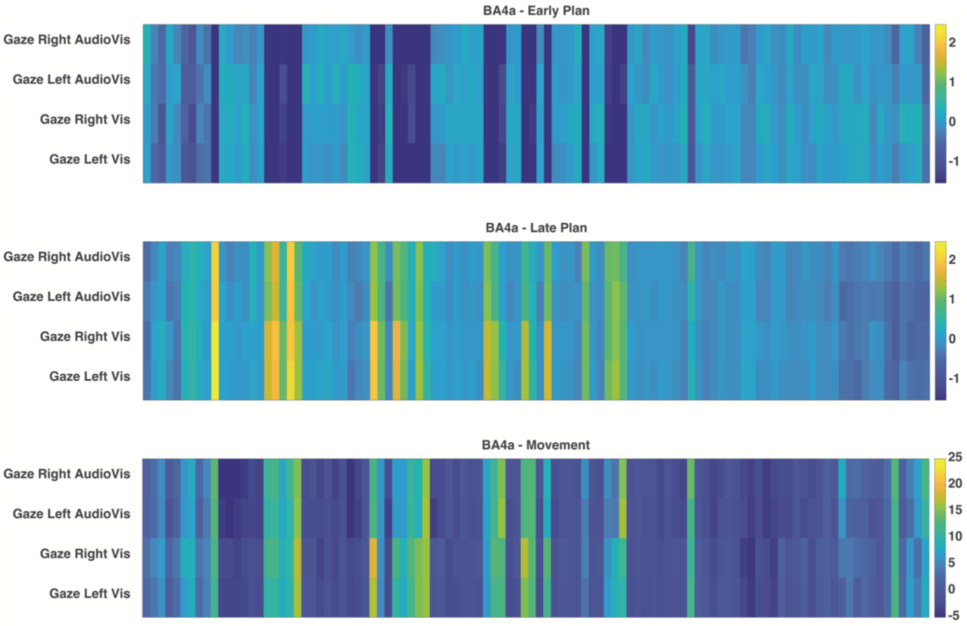

Supplementary Figure 2: General linear model-derived beta distribution maps for the effects of gaze direction and cue modality in region BA4a. Each row in the map is a condition and each column is a voxel in the ROI.

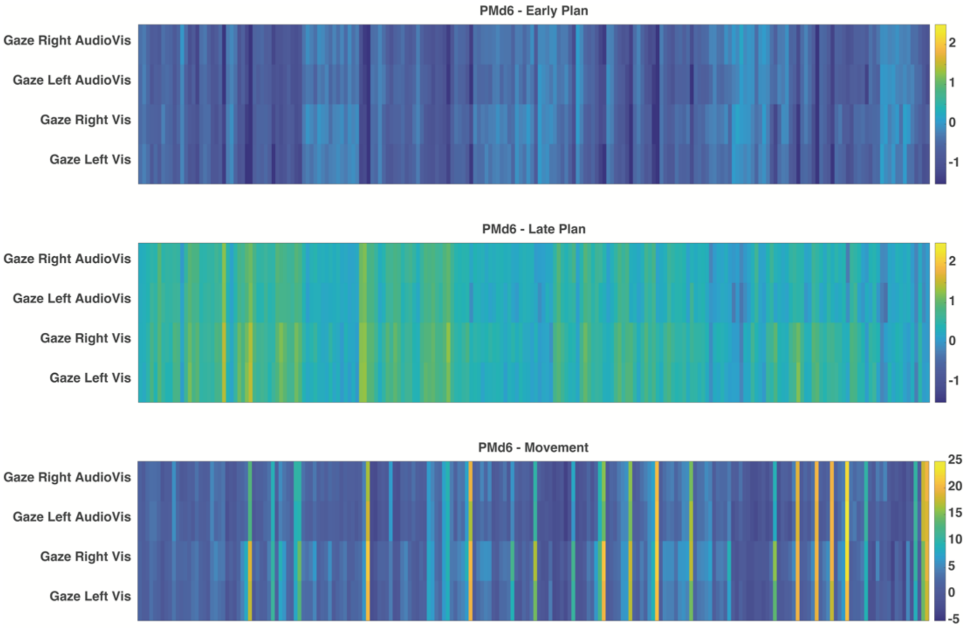

Supplementary Figure 3: General linear model-derived beta distribution maps for the effects of gaze direction and cue modality in region PMd6. Each row in the map is a condition and each column is a voxel in the ROI.

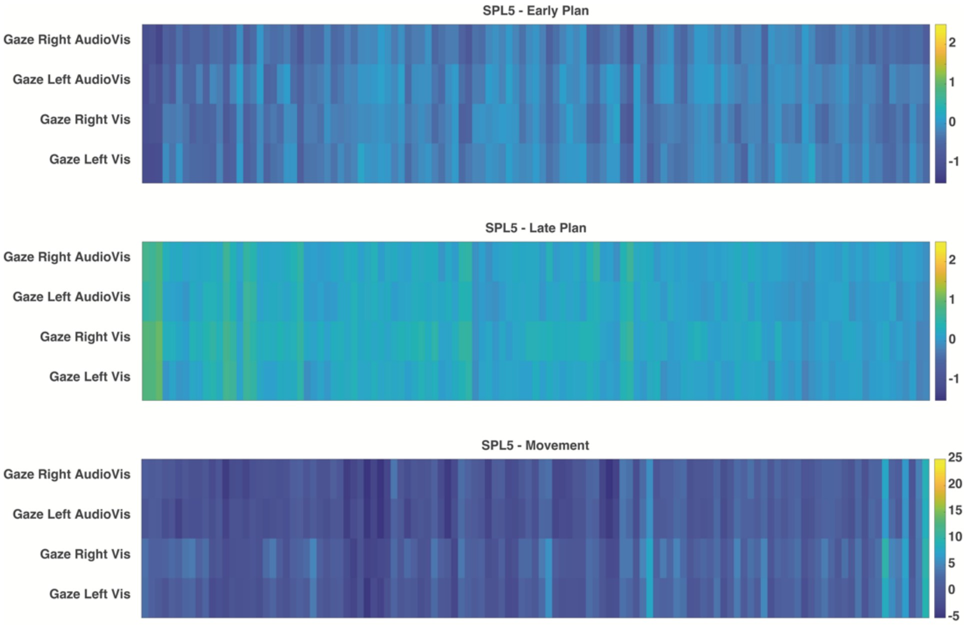

Supplementary Figure 4: General linear model-derived beta distribution maps for the effects of gaze direction and cue modality in region SPL5. Each row in the map is a condition and each column is a voxel in the ROI.

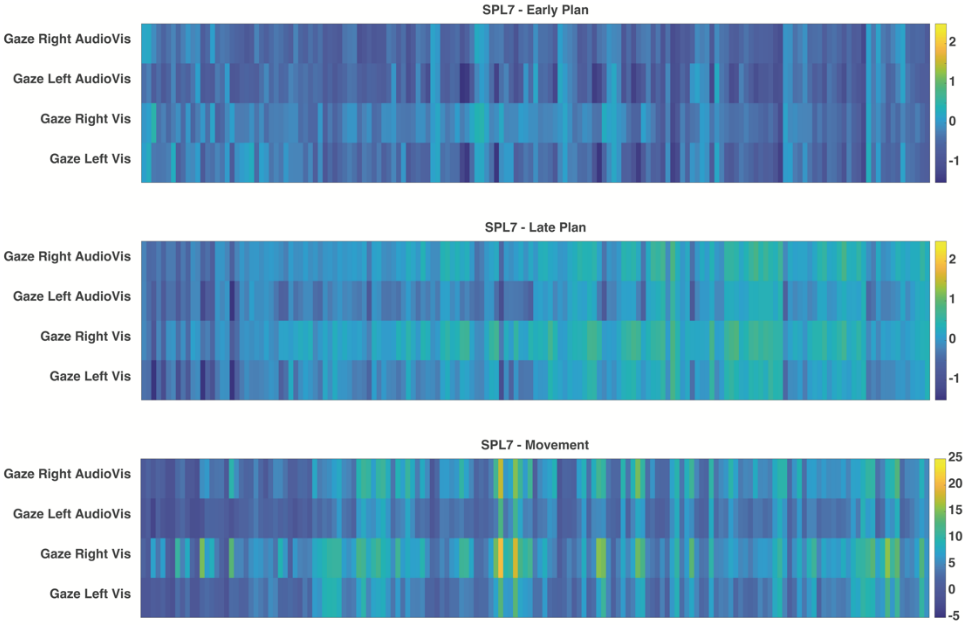

Supplementary Figure 5: General linear model-derived beta distribution maps for the effects of gaze direction and cue modality in region SPL7. Each row in the map is a condition and each column is a voxel in the ROI.

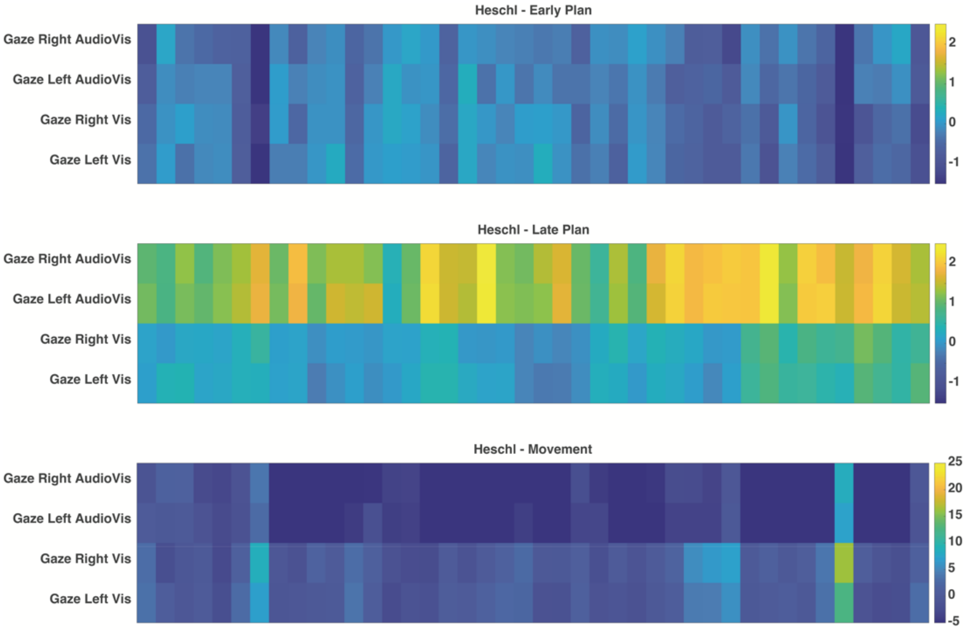

Supplementary Figure 6: General linear model-derived beta distribution maps for the effects of gaze direction and cue modality in Heschl’s gyrus. Each row in the map is a condition and each column is a voxel in the ROI.

b) Effect of Target Direction and Cue Modality – Group Average

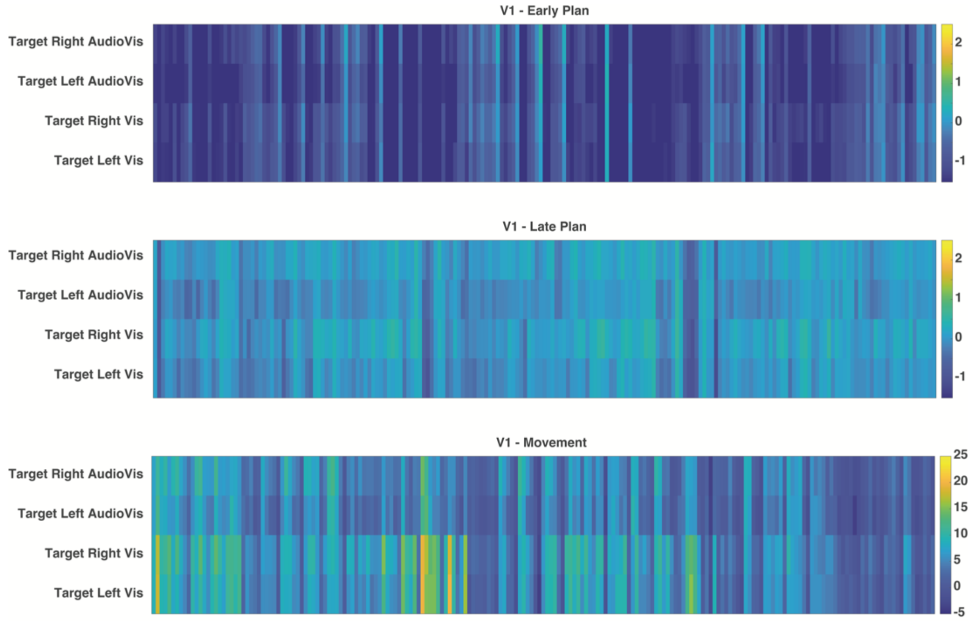

Supplementary Figure 7: General linear model-derived beta distribution maps for the effects of target direction and cue modality in region V1. Each row in the map is a condition and each column is a voxel in the ROI.

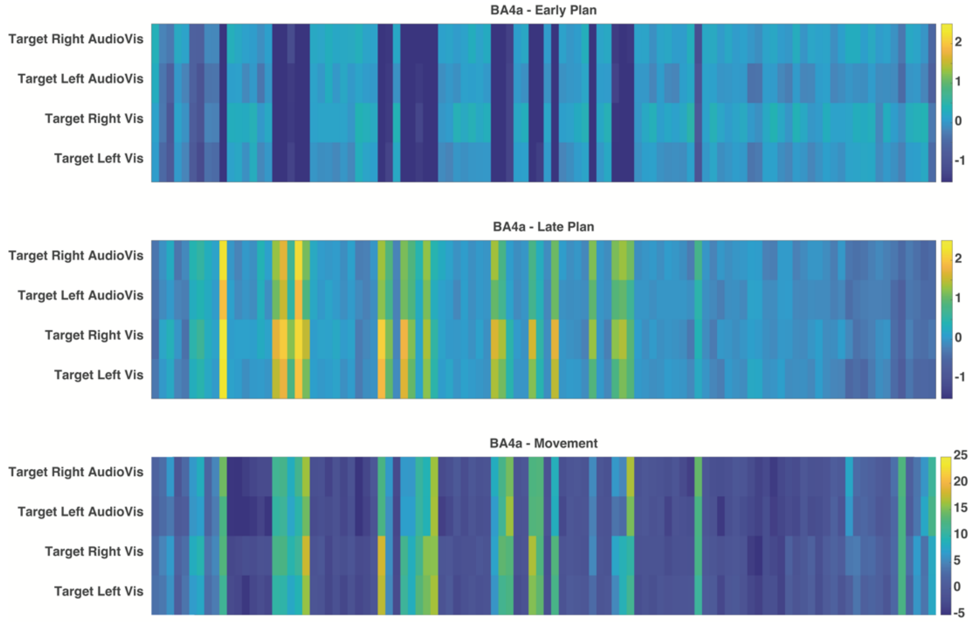

Supplementary Figure 8: General linear model-derived beta distribution maps for the effects of target direction and cue modality in region BA4a. Each row in the map is a condition and each column is a voxel in the ROI.

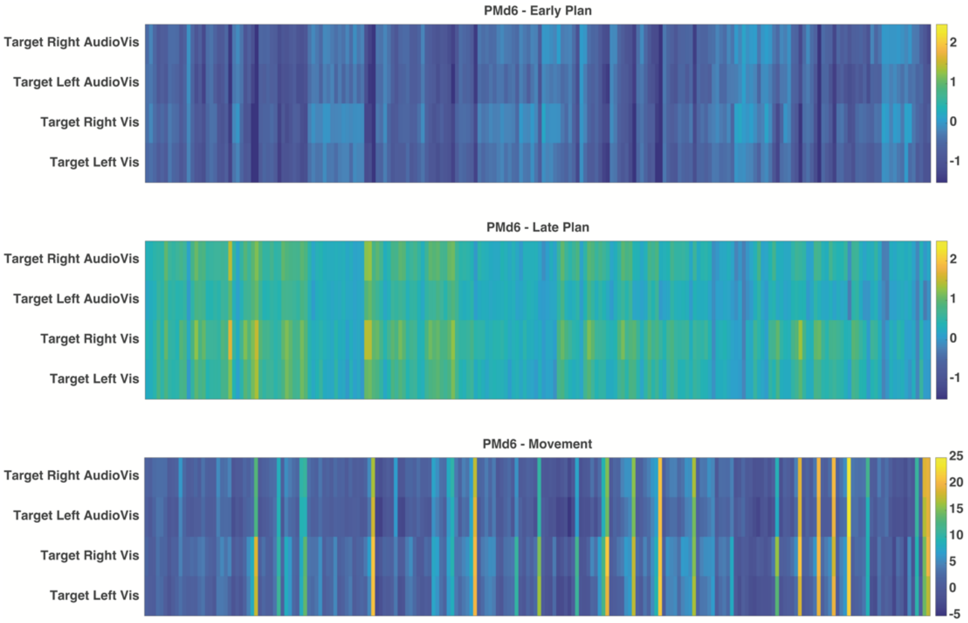

Supplementary Figure 9: General linear model-derived beta distribution maps for the effects of target direction and cue modality in region PMd6. Each row in the map is a condition and each column is a voxel in the ROI.

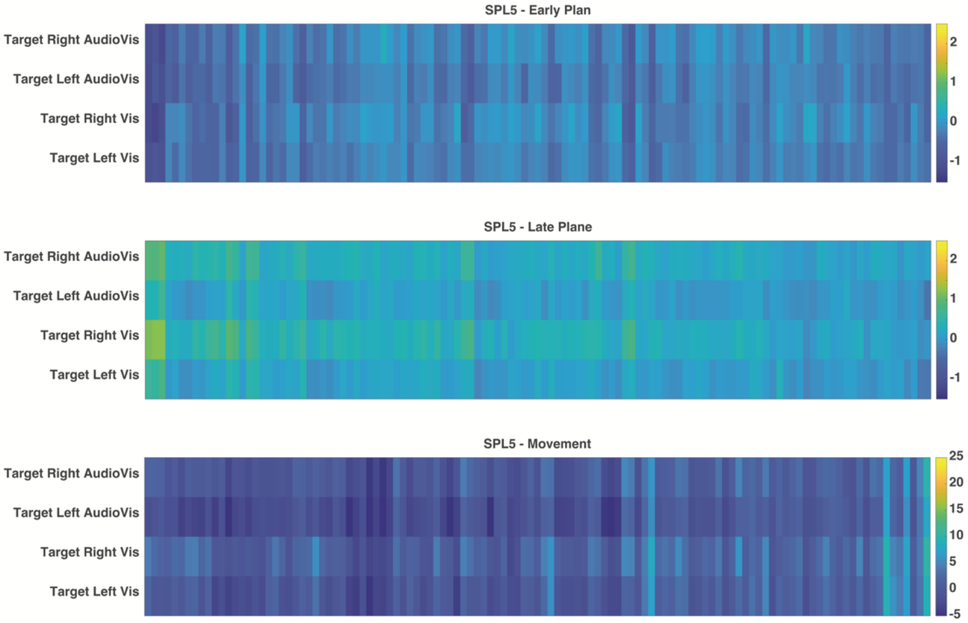

Supplementary Figure 10: General linear model-derived beta distribution maps for the effects of target direction and cue modality in region SPL5. Each row in the map is a condition and each column is a voxel in the ROI.

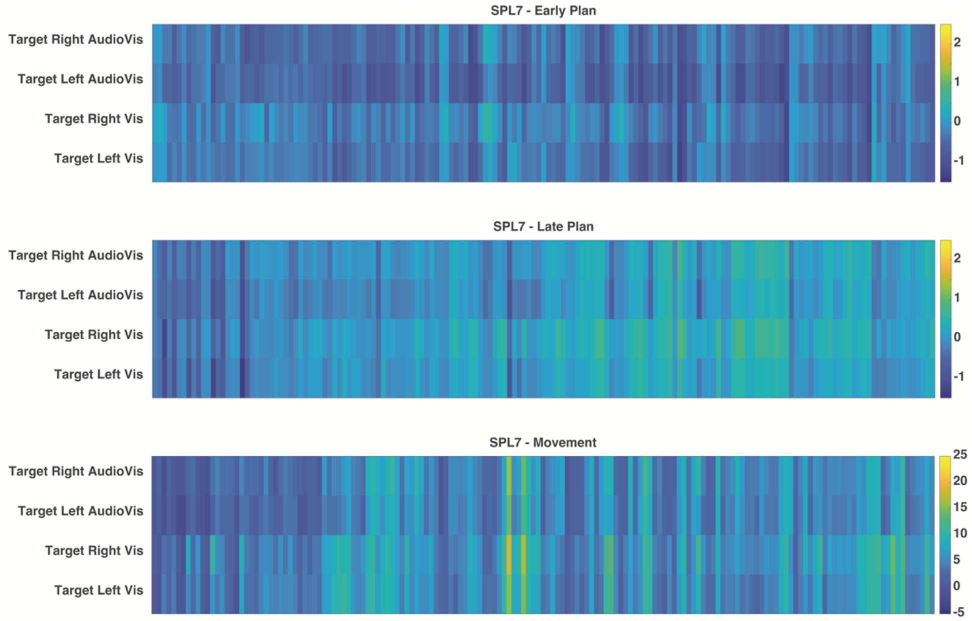

Supplementary Figure 11: General linear model-derived beta distribution maps for the effects of target direction and cue modality in region SPL7. Each row in the map is a condition and each column is a voxel in the ROI.

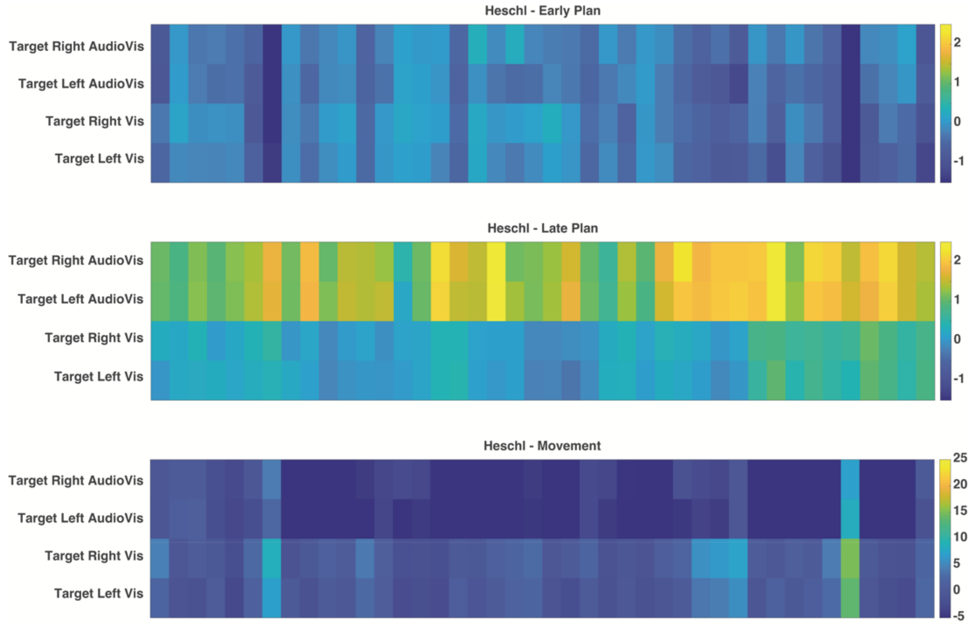

Supplementary Figure 12: General linear model-derived beta distribution maps for the effects of target direction and cue modality in Heschl's gyrus. Each row in the map is a condition and each column is a voxel in the ROI.

c) Effect of Hand Position and Cue Modality – Group Average

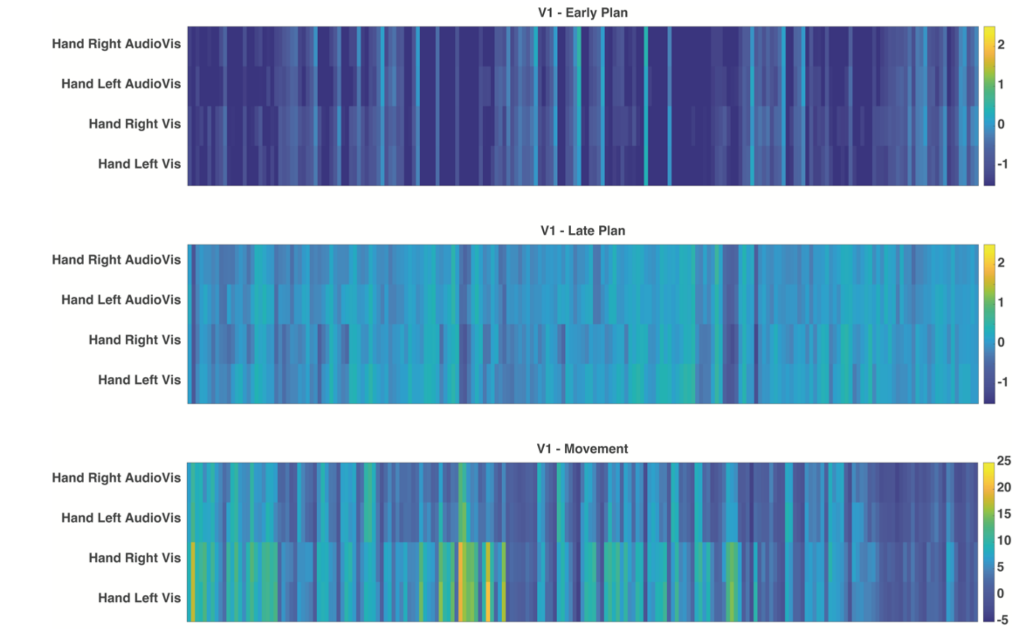

Supplementary Figure 13: General linear model-derived beta distribution maps for the effects of hand position and cue modality in region V1. Each row in the map is a condition and each column is a voxel in the ROI.

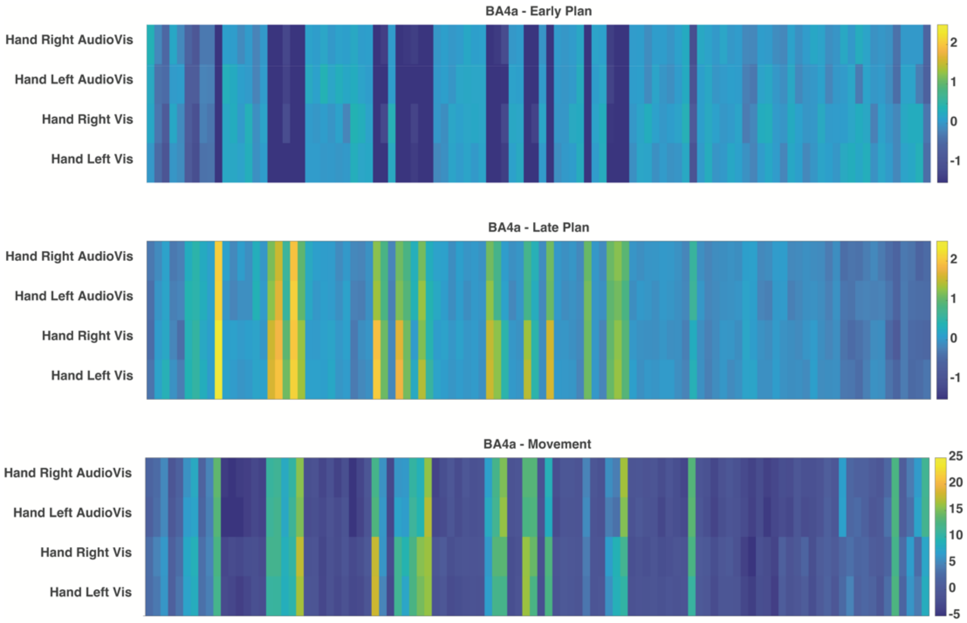

Supplementary Figure 14: General linear model-derived beta distribution maps for the effects of hand position and cue modality in region BA4a. Each row in the map is a condition and each column is a voxel in the ROI.

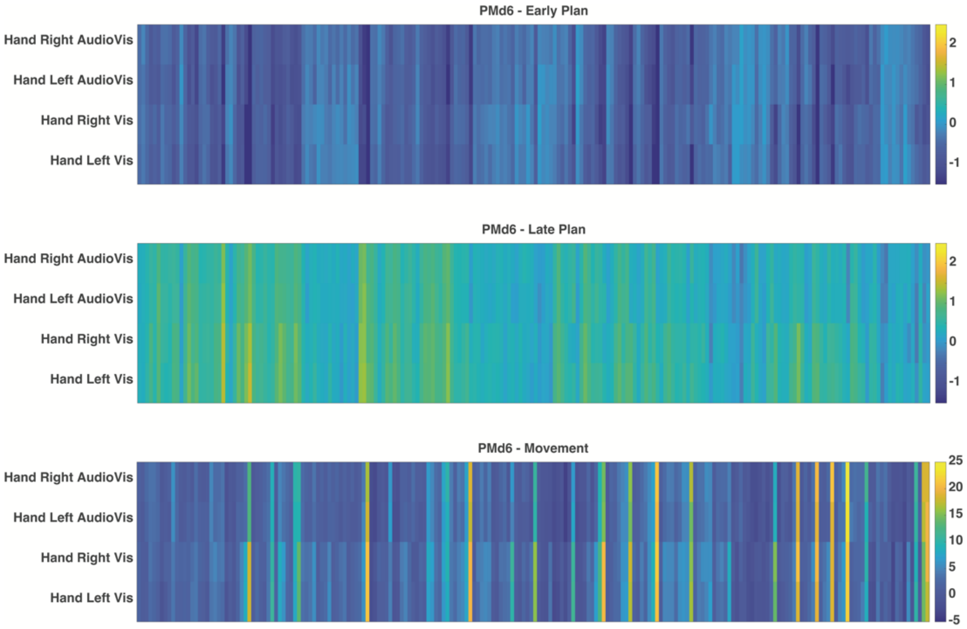

Supplementary Figure 15: General linear model-derived beta distribution maps for the effects of hand position and cue modality in region PMd6. Each row in the map is a condition and each column is a voxel in the ROI.

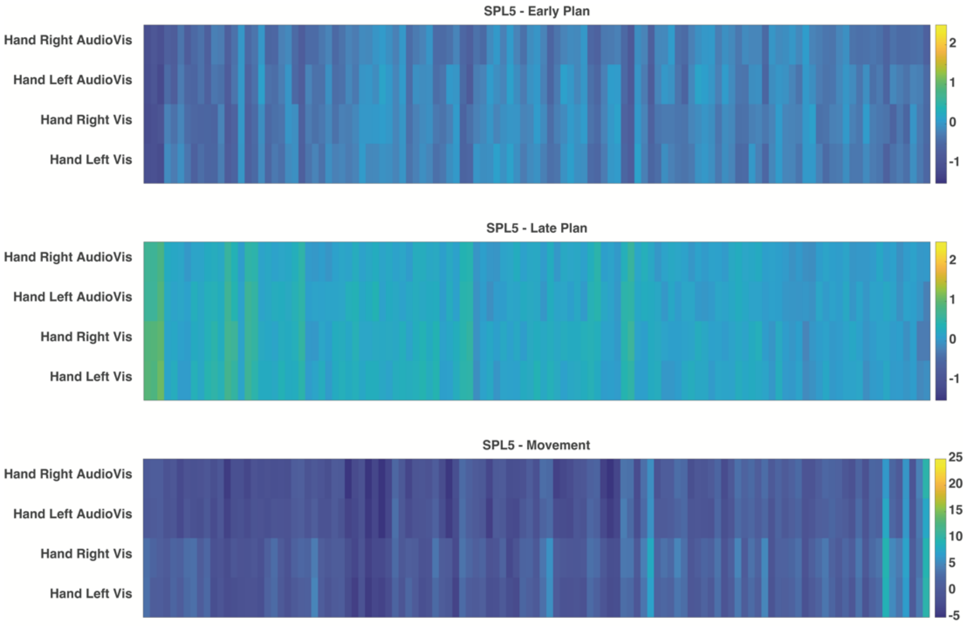

Supplementary Figure 16: General linear model-derived beta distribution maps for the effects of hand position and cue modality in region SPL5. Each row in the map is a condition and each column is a voxel in the ROI.

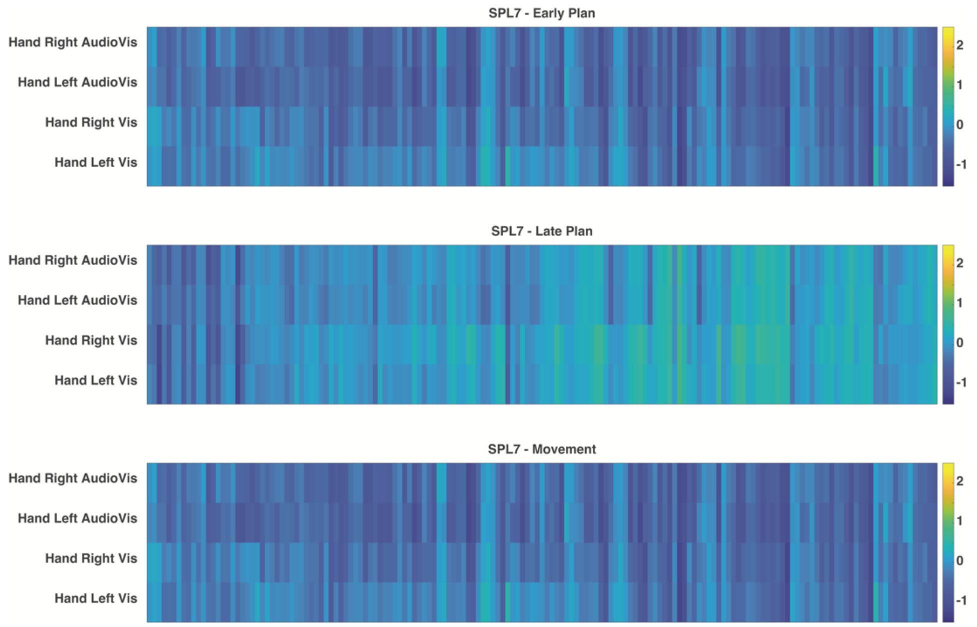

Supplementary Figure 17: General linear model-derived beta distribution maps for the effects of hand position and cue modality in region SPL7. Each row in the map is a condition and each column is a voxel in the ROI.

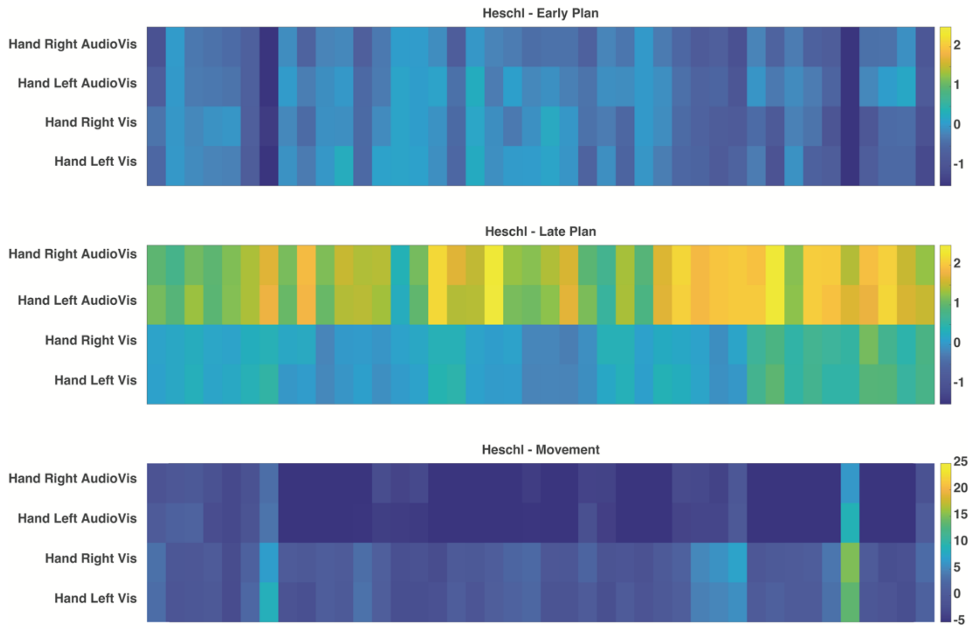

Supplementary Figure 18: General linear model-derived beta distribution maps for the effects of hand position and cue modality in Heschl's gyrus. Each row in the map is a condition and each column is a voxel in the ROI.

d) Effect of Gaze Direction and Cue Modality – Representative Subject

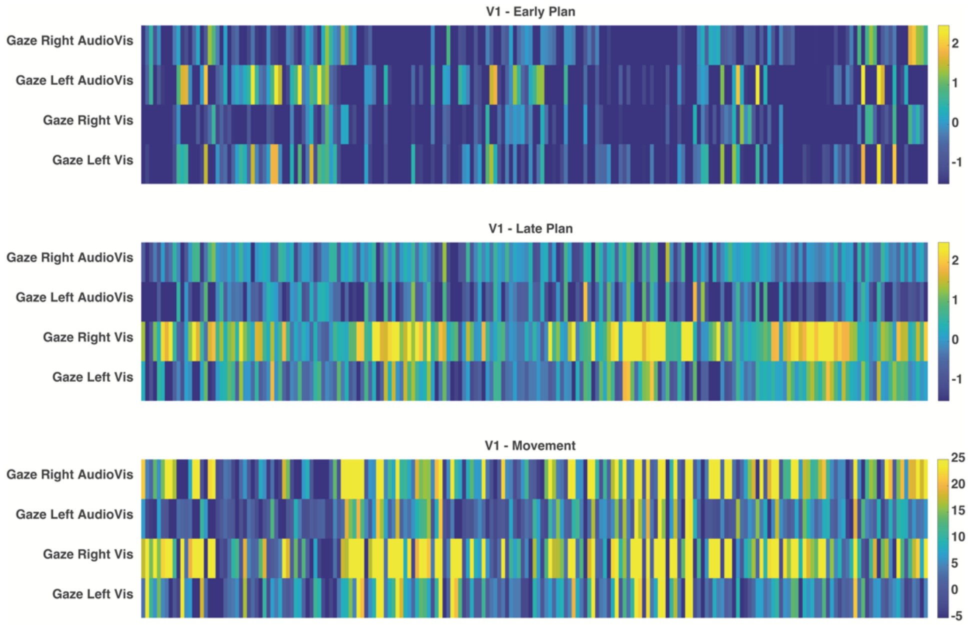

Supplementary Figure 19: General linear model-derived beta distribution maps for the effects of gaze direction and cue modality in region V1. Each row in the map is a condition and each column is a voxel in the ROI.

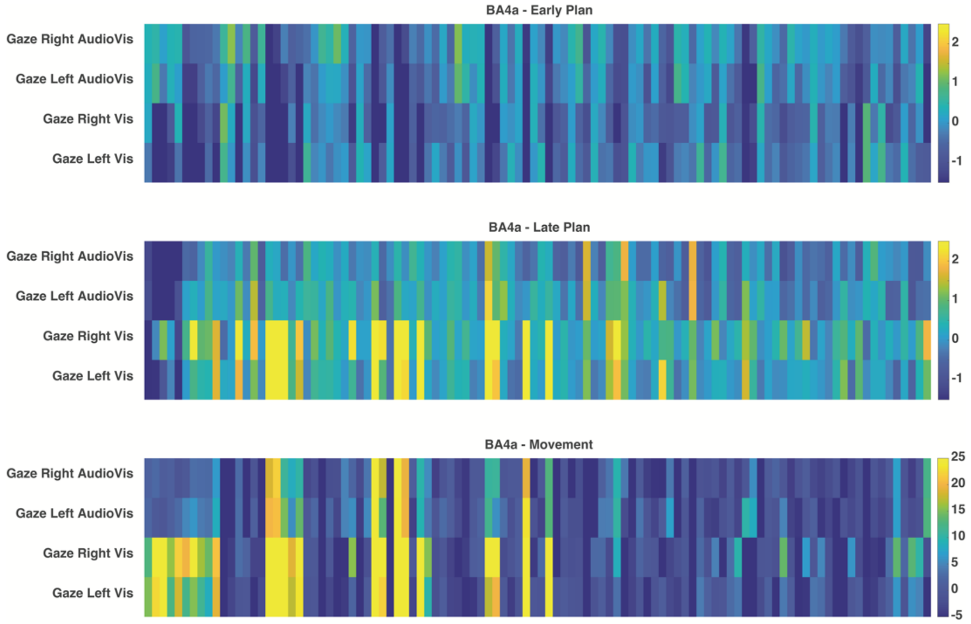

Supplementary Figure 20: General linear model-derived beta distribution maps for the effects of gaze direction and cue modality in region BA4a. Each row in the map is a condition and each column is a voxel in the ROI.

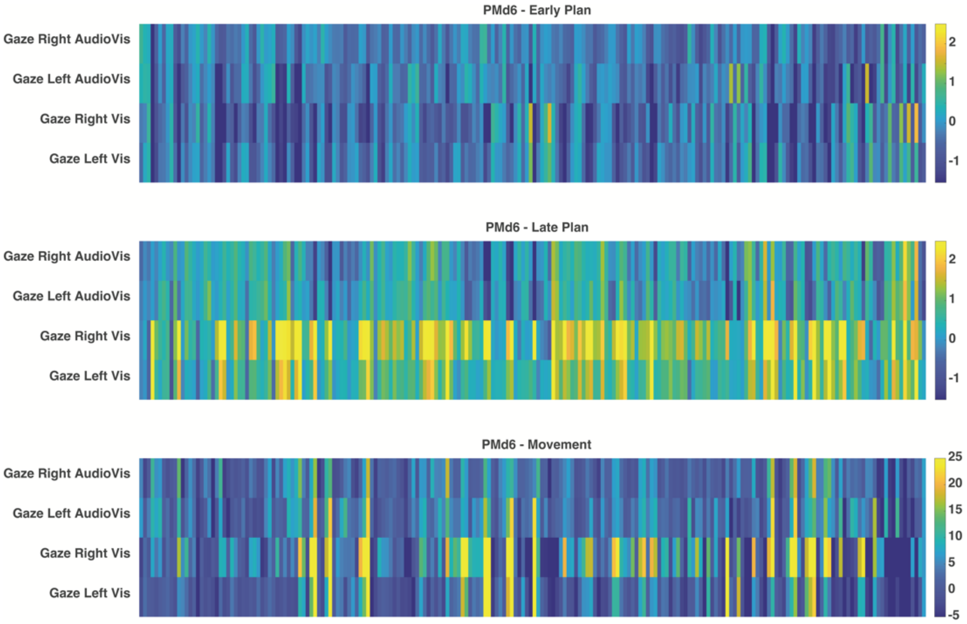

Supplementary Figure 21: General linear model-derived beta distribution maps for the effects of gaze direction and cue modality in region PMd6. Each row in the map is a condition and each column is a voxel in the ROI.

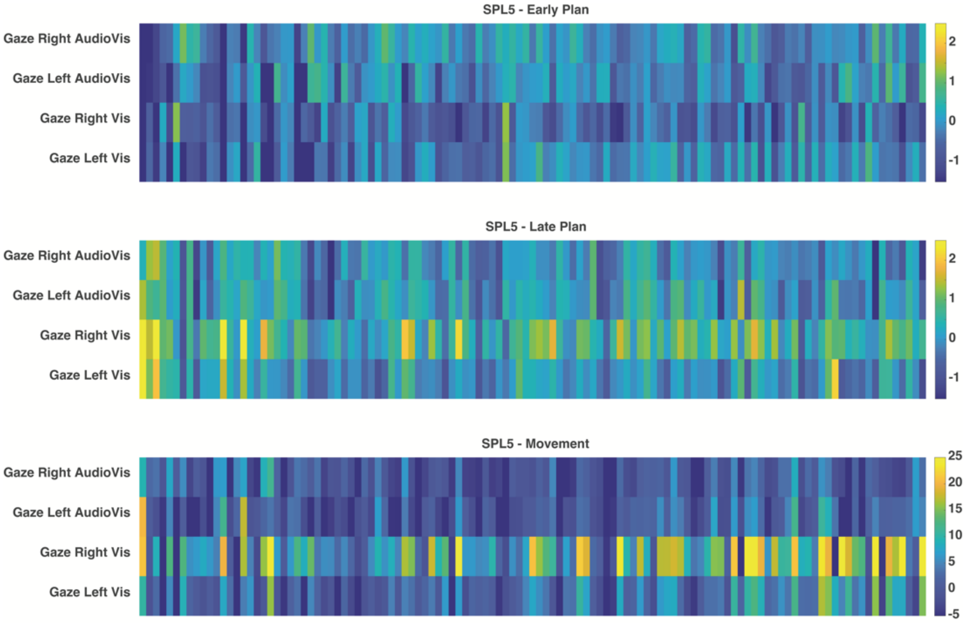

Supplementary Figure 22: General linear model-derived beta distribution maps for the effects of gaze direction and cue modality in region SPL5. Each row in the map is a condition and each column is a voxel in the ROI.

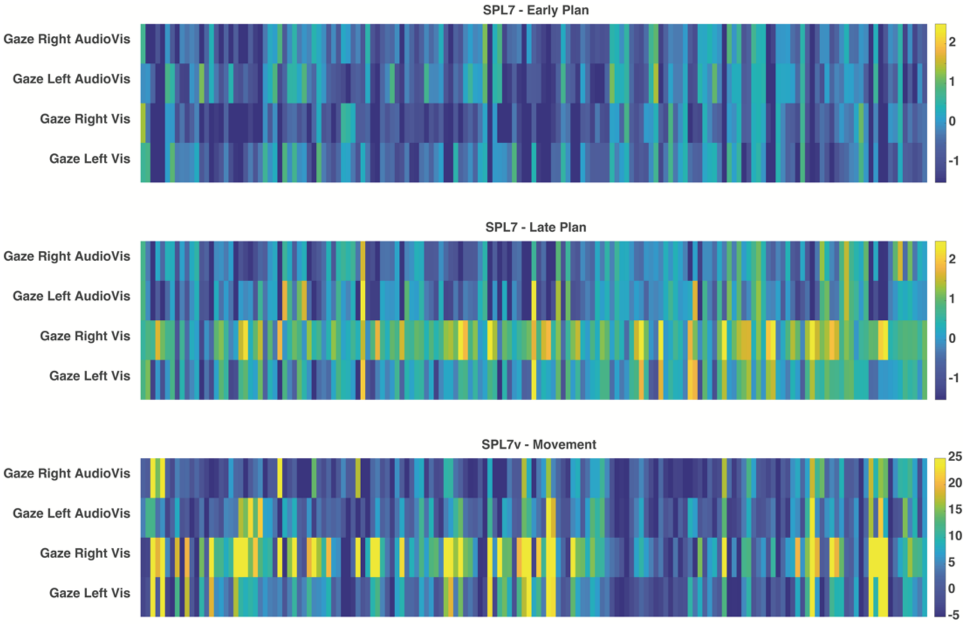

Supplementary Figure 23: General linear model-derived beta distribution maps for the effects of gaze direction and cue modality in region SPL7. Each row in the map is a condition and each column is a voxel in the ROI.

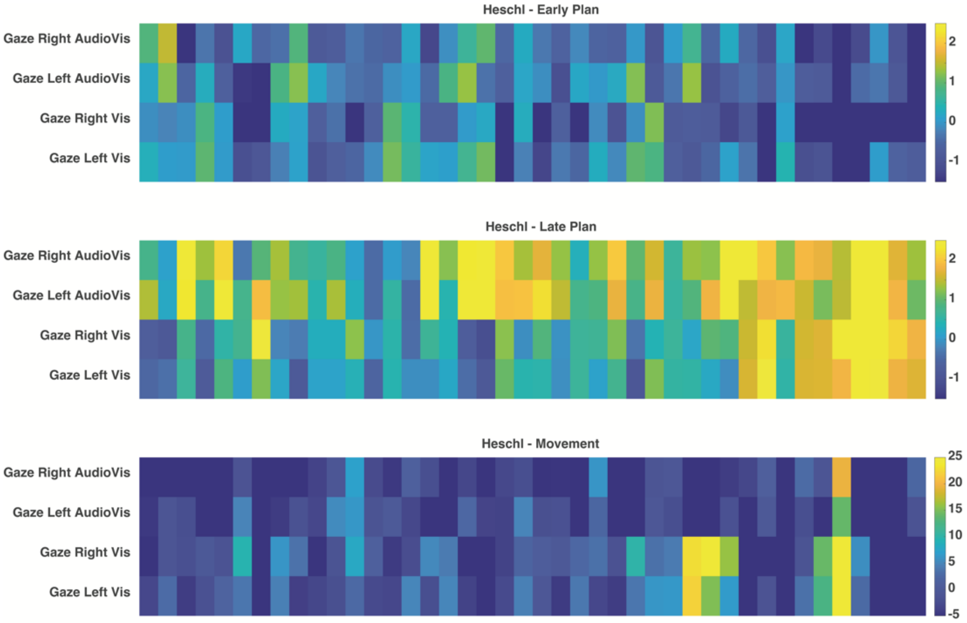

Supplementary Figure 24: General linear model-derived beta distribution maps for the effects of gaze direction and cue modality in Heschl's Gyrus. Each row in the map is a condition and each column is a voxel in the ROI.

e) Effect of Target Direction and Cue Modality – Representative Subject

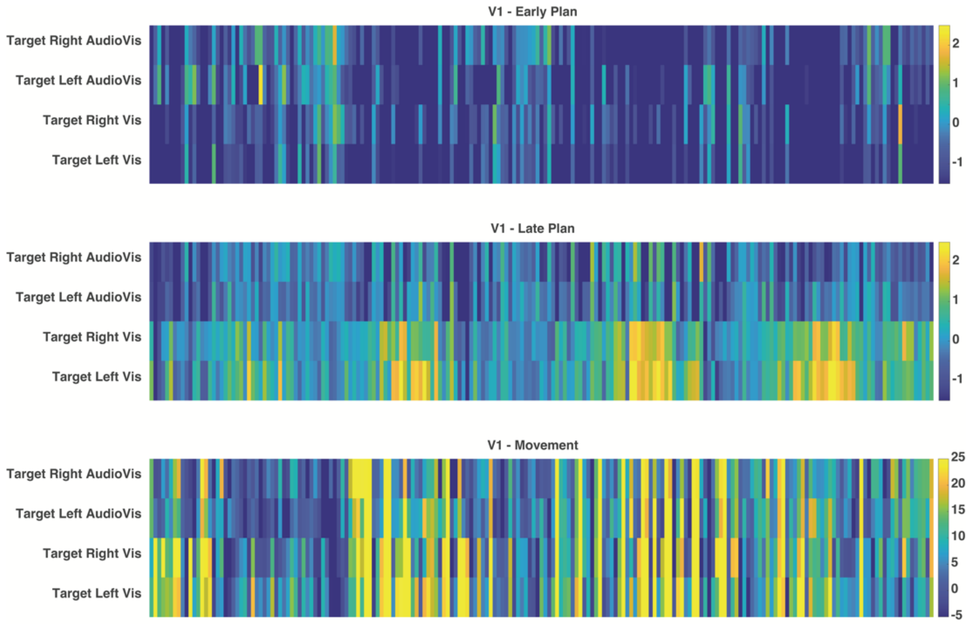

Supplementary Figure 25: General linear model-derived beta distribution maps for the effects of target direction and cue modality in region V1. Each row in the map is a condition and each column is a voxel in the ROI.

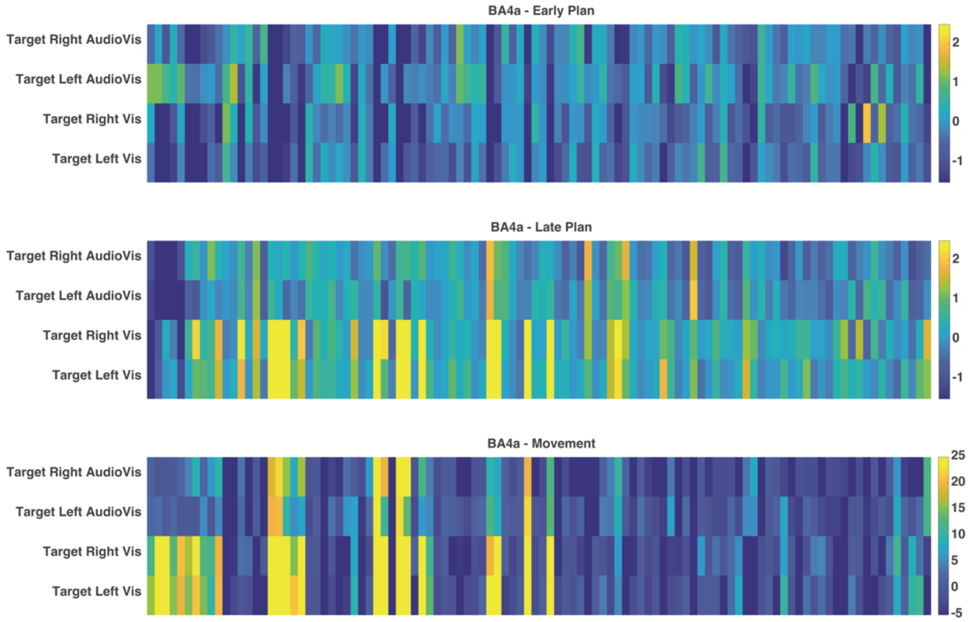

Supplementary Figure 26: General linear model-derived beta distribution maps for the effects of target direction and cue modality in region BA4a. Each row in the map is a condition and each column is a voxel in the ROI.

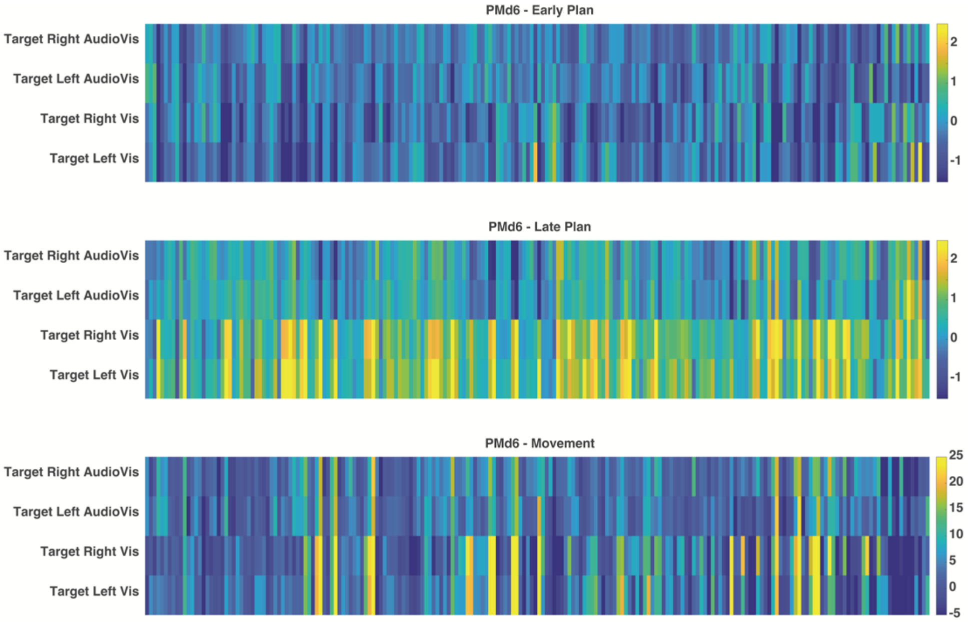

Supplementary Figure 27: General linear model-derived beta distribution maps for the effects of target direction and cue modality in region PMd6. Each row in the map is a condition and each column is a voxel in the ROI.

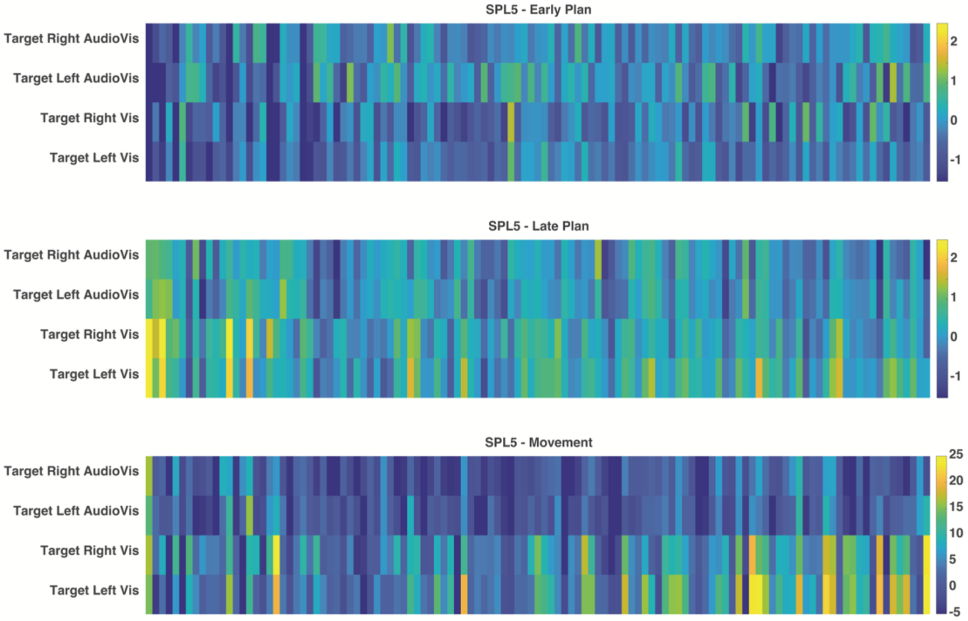

Supplementary Figure 28: General linear model-derived beta distribution maps for the effects of target direction and cue modality in region SPL5. Each row in the map is a condition and each column is a voxel in the ROI.

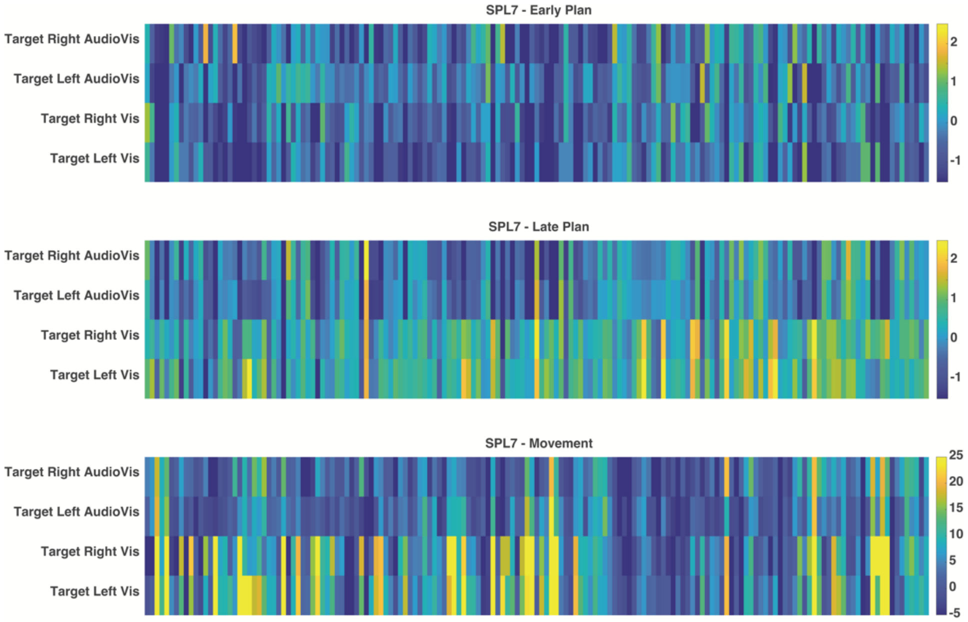

Supplementary Figure 29: General linear model-derived beta distribution maps for the effects of target direction and cue modality in region SPL7. Each row in the map is a condition and each column is a voxel in the ROI.

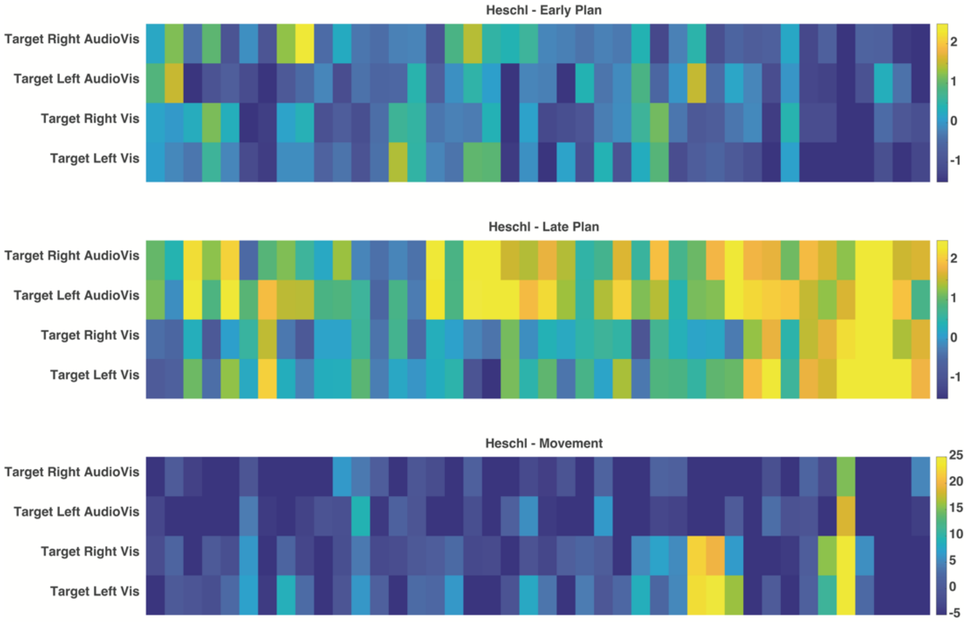

Supplementary Figure 30: General linear model-derived beta distribution maps for the effects of target direction and cue modality in Heschl's Gyrus. Each row in the map is a condition and each column is a voxel in the ROI.

f) Effect of Hand Position and Cue Modality – Representative Subject

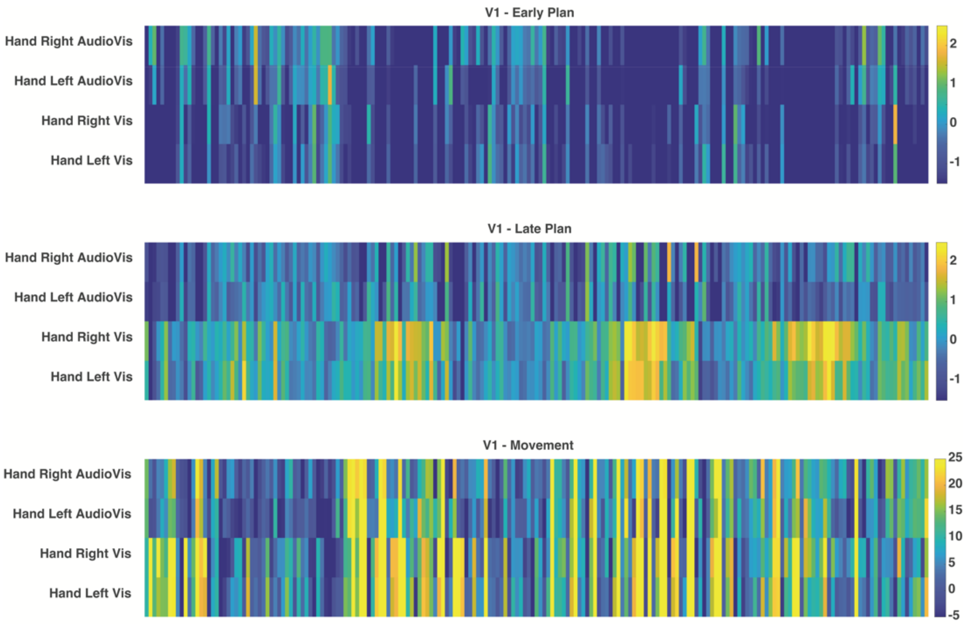

Supplementary Figure 31: General linear model-derived beta distribution maps for the effects of hand position and cue modality in region V1. Each row in the map is a condition and each column is a voxel in the ROI.

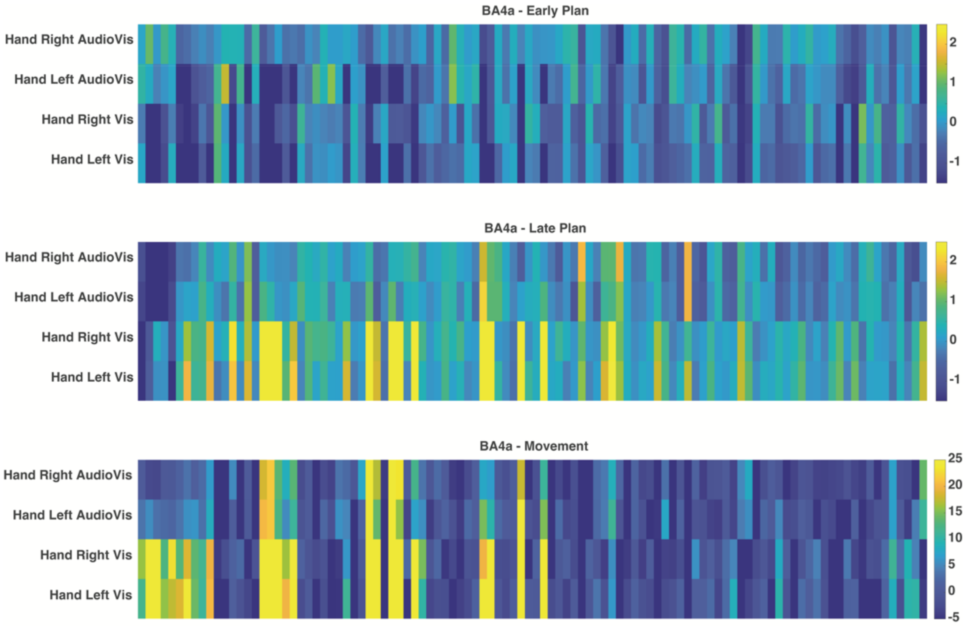

Supplementary Figure 32: General linear model-derived beta distribution maps for the effects of hand position and cue modality in region BA4a. Each row in the map is a condition and each column is a voxel in the ROI.

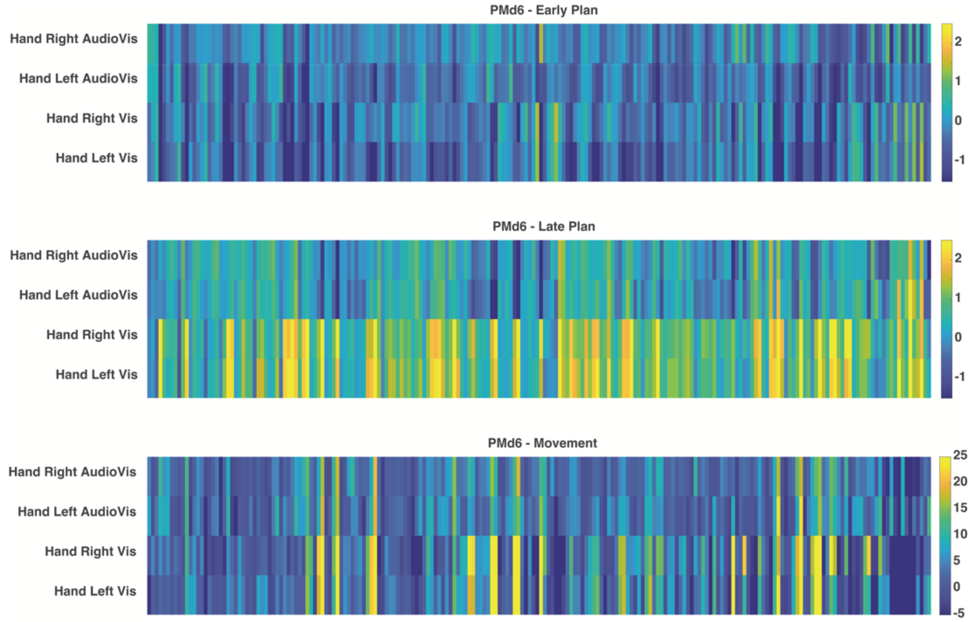

Supplementary Figure 33: General linear model-derived beta distribution maps for the effects of hand position and cue modality in region PMd6. Each row in the map is a condition and each column is a voxel in the ROI.

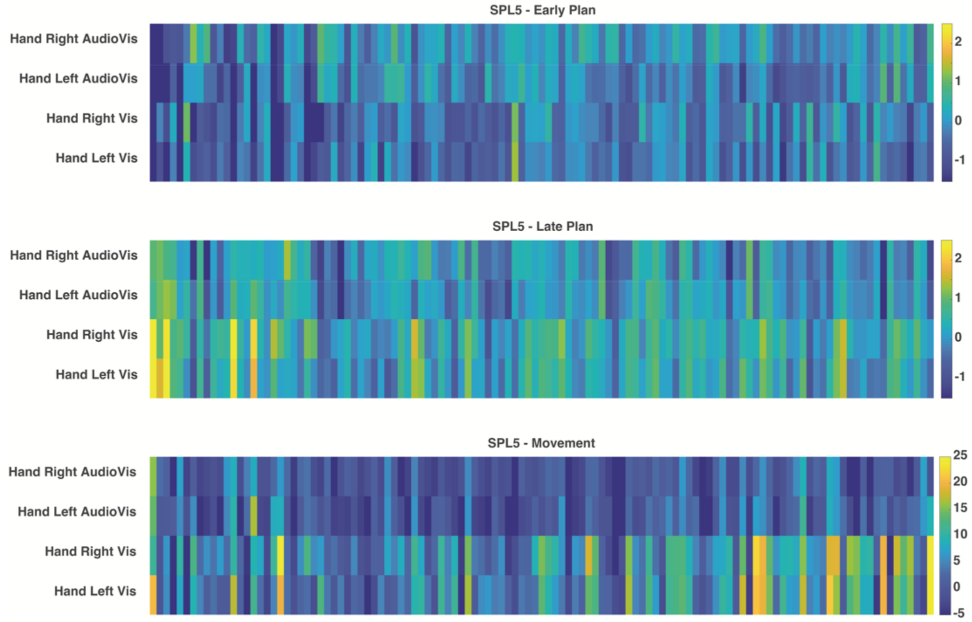

Supplementary Figure 34: General linear model-derived beta distribution maps for the effects of hand position and cue modality in region SPL5. Each row in the map is a condition and each column is a voxel in the ROI.

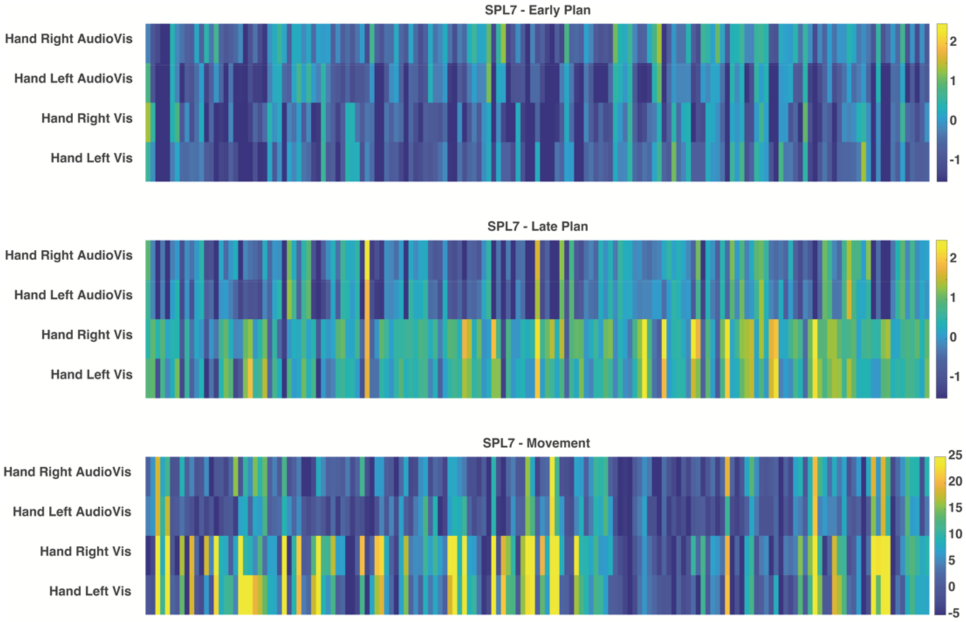

Supplementary Figure 35: General linear model-derived beta distribution maps for the effects of hand position and cue modality in region SPL7. Each row in the map is a condition and each column is a voxel in the ROI.

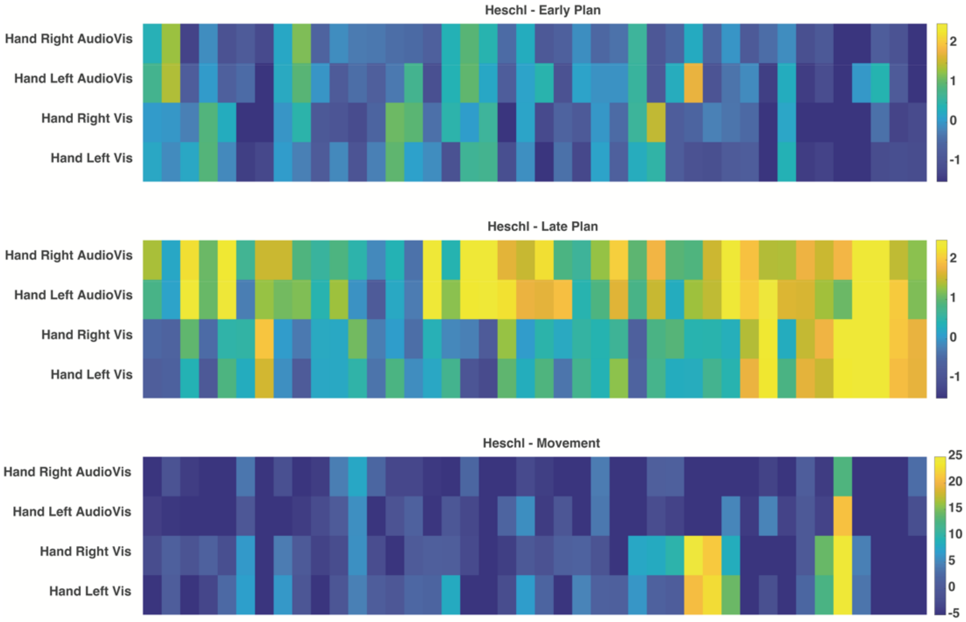

Supplementary Figure 36: General linear model-derived beta distribution maps for the effects of hand position and cue modality in Heschl's Gyrus. Each row in the map is a condition and each column is a voxel in the ROI.

g) Representative G-matrices for Each Epoch

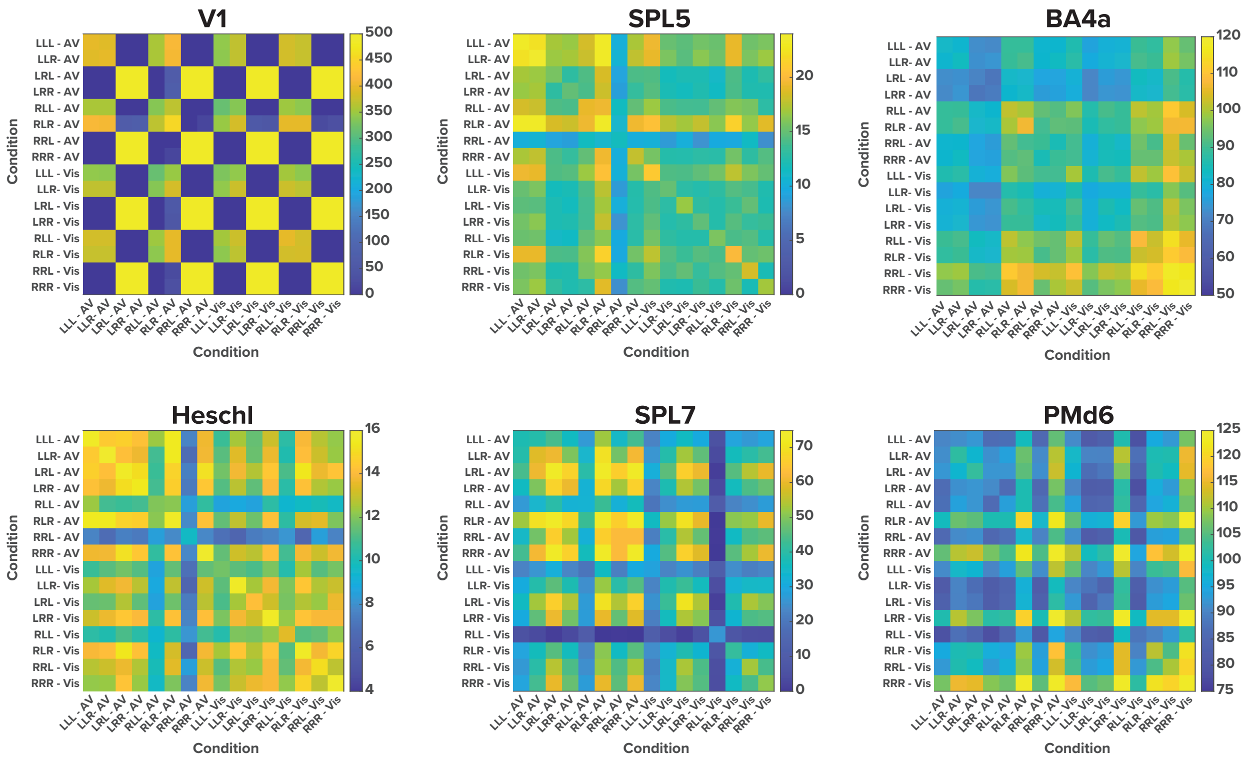

Supplementary Figure 37: G-matrices illustrating the modeled representational structure across conditions in the early planning epoch. The values in the matrices reflect the modeled covariance structure between experimental conditions, providing a visualization of the representational geometry captured by the RSA model. Diagonal elements indicate the modeled variance for each condition, while off-diagonal elements represent the modeled similarity between condition pairs.

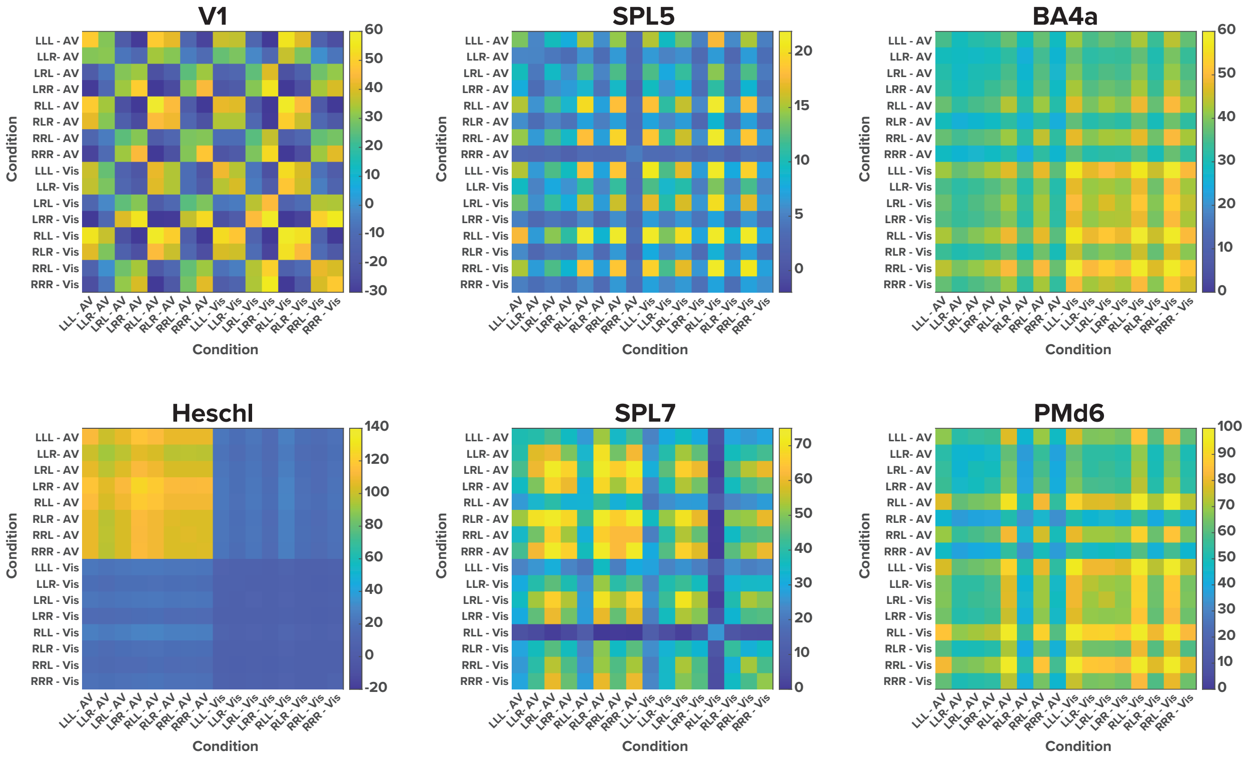

Supplementary Figure 38: G-matrices illustrating the modeled representational structure across conditions in the late planning epoch. The values in the matrices reflect the modeled covariance structure between experimental conditions, providing a visualization of the representational geometry captured by the RSA model. Diagonal elements indicate the modeled variance for each condition, while off-diagonal elements represent the modeled similarity between condition pairs.

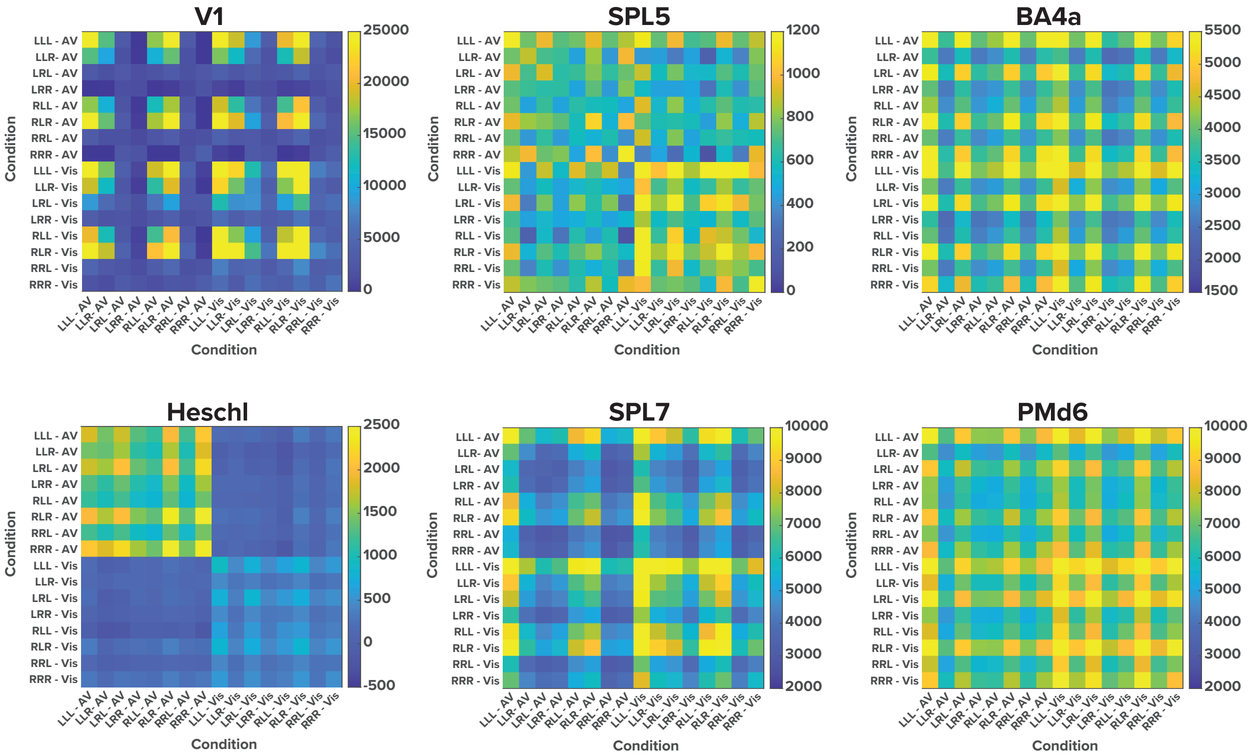

Supplementary Figure 39: G-matrices illustrating the modeled representational structure across conditions in the movement epoch. The values in the matrices reflect the modeled covariance structure between experimental conditions, providing a visualization of the representational geometry captured by the RSA model. Diagonal elements indicate the modeled variance for each condition, while off-diagonal elements represent the modeled similarity between condition pairs.
